# Variation in growth rates of branching corals along Australia’s Great Barrier Reef

**DOI:** 10.1038/s41598-017-03085-1

**Published:** 2017-06-07

**Authors:** Kristen D. Anderson, Neal E. Cantin, Scott F. Heron, Chiara Pisapia, Morgan S. Pratchett

**Affiliations:** 1 ARC Centre of Excellence for Coral Reef Studies, James Cook University, Townsville, QLD 4811 Australia; 2 Australian Institute of Marine Science, PMB 3, Townsville, Queensland 4810 Australia; 3 Coral Reef Watch, U.S. National Oceanic and Atmospheric Administration, College Park, MD 20740 USA; 4 Global Science & Technology, Inc., Greenbelt, MD 20770 USA; 5 Marine Geophysical Laboratory, Physics Department, College of Science and Engineering, James Cook University, Townsville, QLD 4811 Australia

## Abstract

Coral growth is an important component of reef health and resilience. However, few studies have investigated temporal and/or spatial variation in growth of branching corals, which are important contributors to the structure and function of reef habitats. This study assessed growth (linear extension, density, and calcification) of three branching coral species (*Acropora muricata*, *Pocillopora damicornis* and *Isopora palifera*) at three distinct locations (Lizard Island, Davies/Trunk Reef, and Heron Island) along Australia’s Great Barrier Reef (GBR). Annual growth rates of all species were highest at Lizard Island and declined with increasing latitude, corresponding with differences in temperature. Within locations, however, seasonal variation in growth did not directly correlate with temperature. Between October 2012 and October 2014, the highest growth of *A*. *muricata* was in the 2013–14 summer at Lizard Island, which was unusually cool and ~0.5 °C less than the long-term summer average temperature. At locations where temperatures reached or exceeded the long-term summer maxima, coral growth during summer periods was equal to, if not lower than, winter periods. This study shows that temperature has a significant influence on spatiotemporal patterns of branching coral growth, and high summer temperatures in the northern GBR may already be constraining coral growth and reef resilience.

## Introduction

Coral reefs are important ecosystems, providing invaluable goods and services to tropical nations^1^ as well as supporting a great diversity of reef-associated organisms^2–4^. The value and productivity of coral reef ecosystems are strongly linked to the condition of coral assemblages, and especially the structural complexity and habitat diversity provided by coral-rich habitats^5^. Accordingly, widespread declines in coral cover and structural complexity, as reported in a number of locations^6, 7^ directly effect local diversity and productivity^8, 9^, thereby undermining the ecological and economic value of reef ecosystems. Sustained and ongoing declines in abundance of corals are largely attributed to increasing incidence of whole coral mortality caused by acute disturbances^7^. However, acute disturbances (e.g., mass coral bleaching) as well as chronic disturbances (e.g., sustained shifts in environmental conditions) can have important demographic consequences for reef building corals^10, 11^, further contributing to declines in live coral cover.

Coral growth rates are known to be strongly linked to environmental conditions. Individual coral growth is regulated by a wide range of both biotic and abiotic factors^12^. However, at large latitudinal scales, aragonite saturation state, temperature, and light control the geographic distribution of corals, as they are critical factors for photosynthesis (energy acquisition) and calcification^13^. Beyond the latitudinal limit of reef formation (~30°), aragonite saturation is generally too low (Ω_arag_ < 3) to enable reef accretion and formation^14^. At lower latitudes coral growth generally increases in accordance with increasing temperature, carbonate saturation and light intensity^12^. Across 14 degrees of latitude on Australia’s Great Barrier Reef (GBR) (10 °S to 24 °S), an increase of 1 °C in annual average sea surface temperature (SST) was correlated with an increase in calcification rates by 0.3 g cm^−2^ year^−1^ for massive *Porites* ^15^.

Despite the generally positive relationship between coral growth and temperature, ocean warming is of great concern for scleractinian corals^16, 17^. Most notably, corals may bleach when the local temperatures exceed typical summer maxima by 1 °C^18^. However, even before corals bleach sustained increases in local temperatures may lead to declines in growth or performance. For example, the optimum temperature for coral calcification is typically 1–3 °C below the local summer maximum^19, 20^, such that ocean warming will constrain coral growth by reducing the time that environmental conditions are conducive to maximum rates of calcification^12^. Ocean warming is purported to be key factor contributing to observed declines in growth of massive corals across many reef locations (Australia^21^, Thailand^22^, Red Sea^23^, Florida^24^).

While the effects of ocean warming on coral growth are relatively well understood, current knowledge regarding projected changes in aragonite saturation (Ω_arag_) on coral reefs and how this will effect demography of scleractinain corals is very limited^25^. Increases in atmospheric carbon dioxide (CO_2_) from 280 ppm in the pre-industrial period to present day concentrations of 401 ppm^26^ have resulted in a decrease in the surface ocean pH from a global average of 8.21 to 8.10, as well as corresponding shifts in aragonite saturation^27^. As the aragonite saturation declines, the carbonate saturation state of the internal calcifying fluid declines and the rate of calcification decreases^28^. Albright *et al*.^29^ used alkalinity enrichment to demonstrate that net community calcification increases significantly when ocean chemistry is restored to conditions expected to have occurred in pre-industrial times.

**Figure 1:**
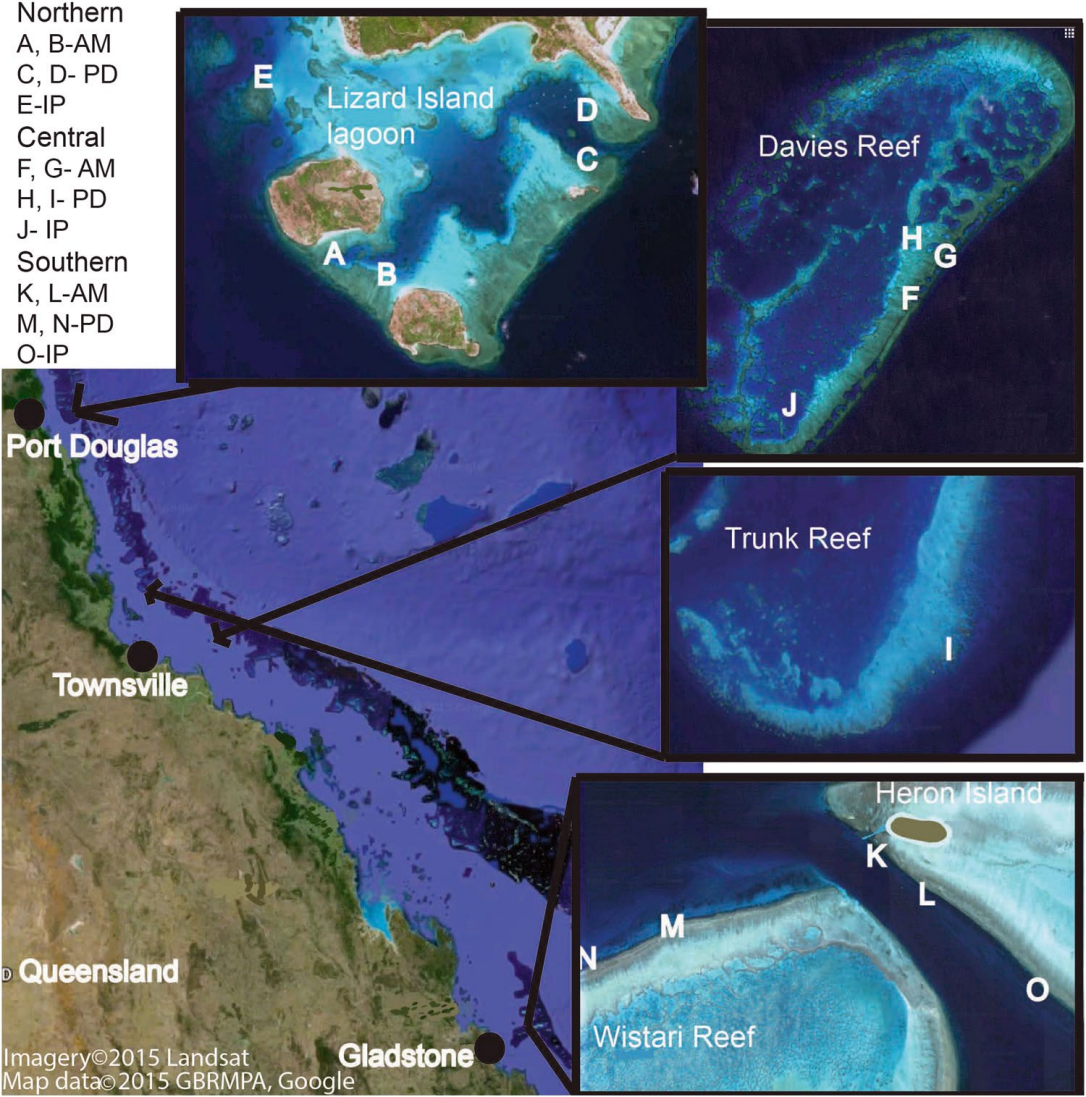
Map of study sites along the Great Barrier Reef. Sites A,B (*A*. *muricata*), C,D (*P*. *damicornis*), E (*I*. *palifera*) were in the northern sector at Lizard Island. Sites F, G (*A*. *muricata*), H, I, (*P*. *damicornis*), J (*I*. *palifera*) were in the central sector at Davies and Trunk Reef. Sites K, L, (*A*. *muricata*), M, N (*P*. *damicornis*), O (*I*. *palifera*) were in the southern sector at Heron Island. AM = *Acropoca muricata*. PD = *Pocillopora damicornis*. IP = *Isopora palifera*. Figure data provided by Imagery©2016 Landsat, Data SIO, NOAA, U.S. Navy, NGA, GEBCO, Map data ©2015 GBRMPA, Google 2015.

**Figure 2:**
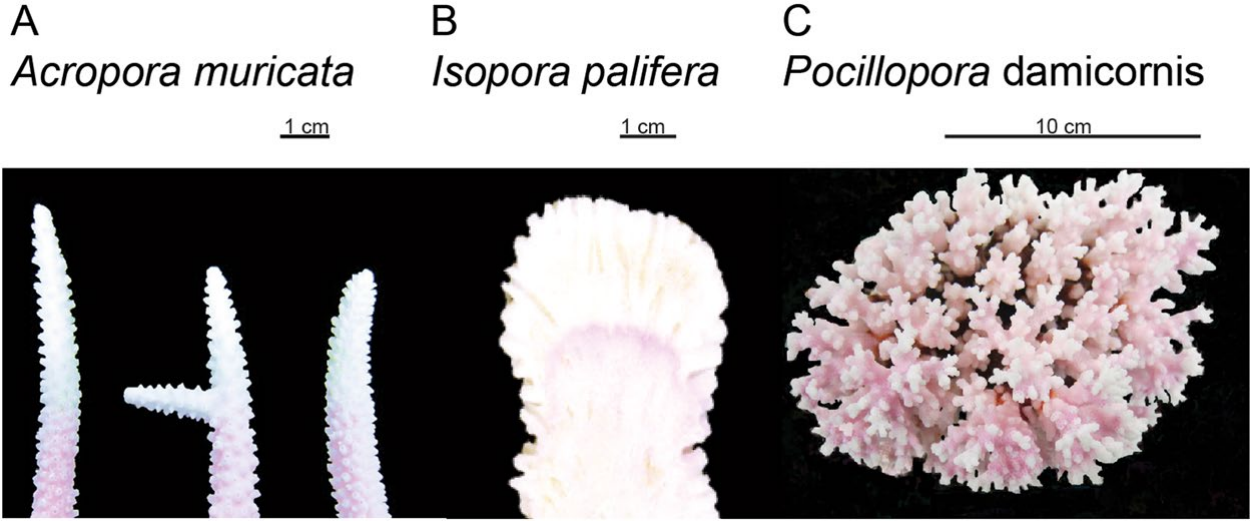
Coral species (**A**) *Acropora muricata*, (**B**) *Isopora palifera* and (**C**) *Pocillopora damicornis* utilized in this study. The pink portion of the skeleton is the result of the Alizarin Red dye being incorporated into the skeleton from staining and the white portion is the newly accreted skeleton.

The purpose of this study was to quantify growth rates (specifically, linear extension, density and calcification) of three branching corals (*Acropora muricata* (cf. *A*. *formosa*), *Pocillopora damicornis* and *Isopora palifera*) at three distinct locations along the Great Barrier Reef, Australia (Figs 1 and 2). Until recently, much of the work on changes in coral growth rates along marked environmental gradients and the documented effects of climate change have focused on massive coral species, largely due to relative ease in documenting coral growth rates from the alternating high and low density banding^12^. The spatial trends in growth rates of most branching coral species are unknown and yet they are arguably the most ecologically important corals in providing reef complexity, and therefore, reef biodiversity^30^. Declines in the growth rates of branching corals, which are among the fastest growing corals, may also have significant impacts on reef growth^12^. Investigating variability in growth of branching corals over large temporal and spatial scales, where there are substantial differences in environmental conditions may provide insight into how these corals will respond to changing environmental conditions.

## Results

Staining of branching corals to monitor growth is a common method, however some loss of stained branches and colonies is inevitable. Moderate levels of background coral mortality and injuries are common for fast growing and typically short-lived, branching corals^31^. Furthermore, despite return visits to study sites every six or twelve months, several stained colonies could not be found. Of the 720 *A*. *muricata* branches stained in this study, 70% (540/720) were recovered and healthy, and were used in analyses of coral growth. Of those excluded, 20% had died since staining but were still intact, while the remainder were not found. For *P*. *damicornis*, 90% (108/120) of the colonies stained were found healthy and used in this study. Of the 12 colonies not included, 7 died, 4 were not found and 1 did not stain well. For *Isopora palifera*, 90% (27/30) of stained colonies were found healthy and used in the study, while the fate of the other 10% is unknown (possibly lost due to poor tag retention).

### Intraspecific variation in growth

#### *Acropora muricata*

**Figure 3:**
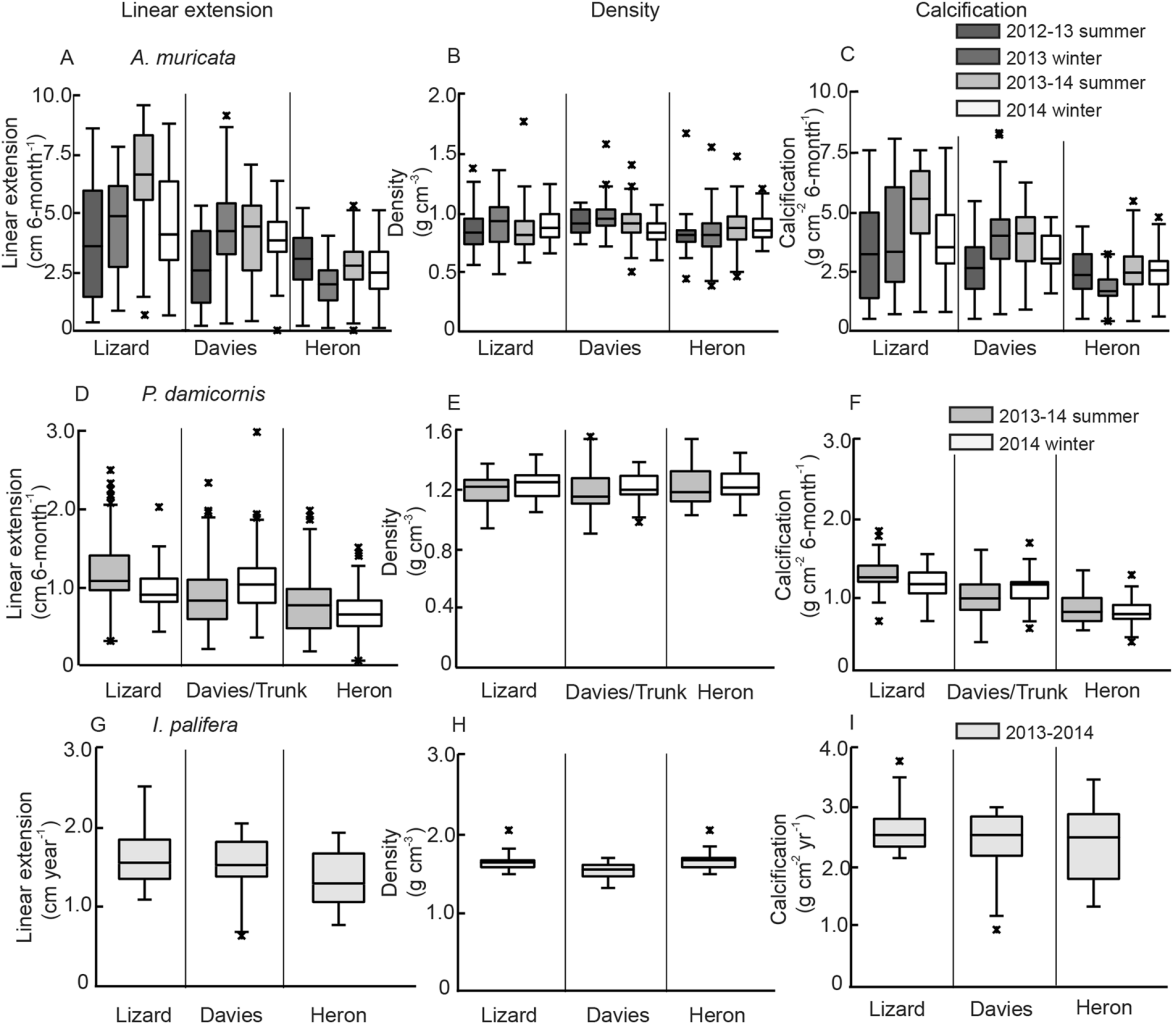
Variation in linear extension (**A**,**D**,**G**), density (**B**,**E**,**H**) and calcification (**C**,**F**,**I**) of *Acropora muricata* (**A**,**B**,**C**), *Pocillopora damicornis* (**D**,**E**,**F**) and *Isopora palifera* (**G**,**H**,**I**) at Lizard Island in the north, Davies Reef and/or Trunk Reef in the center, and Heron Island in the south sectors of the Great Barrier Reef.

Average linear extension was greatest at Lizard Island (4.78 ± 0.19 cm 6-month^−1^) (mean ± SE) compared to Davies Reef (3.81 ± 0.42 cm 6-month^−1^) and Heron Island (2.49 ± 0.08 cm 6-month^−1^). Linear extension rates measured at Lizard Island were significantly greater than Heron Island (lme, t = −3.390, p = 0.003; Supplementary Table S2), but were not significantly different from Davies Reef (lme, t = −1.250, p = 0.229). When evaluating variation among sampling periods within each reef, linear extension showed significant inter-annual variation (Nested ANOVA p < 0.05; Supplementary Table S3). At Lizard Island for example, the lowest (3.69 ± 0.43 cm 6-months^−1^) and greatest (6.56 ± 0.43 cm 6-months^−1^) linear extension rates were in the 2012–13 summer and 2013–14 summer, respectively (Fig. 3A).

The average bulk skeletal density of *A*. *muricata* on the Great Barrier Reef was 0.88 ± 0.01 g cm^−3^, and did not vary significantly among locations (Fig. 3B; lme, p > 0.05, Supplementary Table S2). There was, however, significant temporal variation in density of newly accreted skeleton at Davies Reef between sampling periods (Nested ANOVA, F_3/45_ = 3.319, p = 0.028, Supplementary Table S3) but no consistent trend with respect to season (Fig. 3B).

Calcification rates of *A*. *muricata* (measured as total weight per volume of skeletal material added at growing tips, i.e. the product of branch tip density and linear extension) decreased along the north-south latitudinal gradient along the GBR and were largely reflective of variation in linear extension (Supplementary Fig. S2). The average 6-month calcification rate at Heron Island (2.20 ± 0.4 g cm^−2^) was 40% less than Lizard Island (3.70 ± 0.4 g cm^−2^) and significantly varied (lme, t = −3.280, p = 0.004; Supplementary Table S2). Similar significant variation in calcification (34% less) was observed between Davies Reef (3.31 ± 0.5 g cm^−2^) and Heron Island (Fig. 3C; lme, t = −2.47, p = 0.024). However, calcification rates between Lizard and Davies were comparable (lme, t = −0.759, p = 0.458; Supplementary Table S2). Within each reef, there was significant variation in calcification rates (Nested ANOVA p < 0.05; Supplementary Table S3, Fig. 3C). Annual rates of coral growth are often compared among species^12^ and calculated annual rates of linear extension, density and calcification from this study are provided in Table 1.

**Table 1:** Summary of the annual growth parameters, linear extension (cm yr^−1^), density (g cm^−3^), and calcification (g cm^−2^ yr^−1^) (Mean ± SE), for *A*. *muricata*, *P*. *damicornis and I*. *palifera* at Lizard Island, Davies/Trunk Reefs and Heron Island from 2012/13 and 2013/14.

Species	Location	Year	Linear Extension (cm yr^−1^)	Density (g cm^−3^)	Calcification (g cm^−2^ yr^−1^)
*Acropora muricata*	Lizard Island	2012/13	7.93 ± 0.29	0.89 ± 0.02	7.04 ± 0.29
		2013/14	10.8 ± 0.24	0.87 ± 0.02	9.43 ± 0.22
	Davies Reef	2012/13	7.34 ± 0.24	0.95 ± 0.02	6.95 ± 0.27
		2013/14	7.93 ± 0.21	0.89 ± 0.05	6.97 ± 0.16
	Heron Island	2012/13	4.78 ± 0.12	0.82 ± 0.02	3.93 ± 0.09
		2013/14	5.17 ± 0.21	0.88 ± 0.04	4.57 ± 0.12
*Pocillopora damicornis*	Lizard Island	2013/14	2.20 ± 0.17	1.22 ± 0.03	2.68 ± 0.05
	Davies/Trunk Reef	2013/14	1.93 ± 0.17	1.18 ± 0.03	2.28 ± 0.05
	Heron Island	2013/14	1.50 ± 0.15	1.23 ± 0.02	1.85 ± 0.04
*Isopora palifera*	Lizard Island	2013/14	1.64 ± 0.35	1.66 ± 0.03	2.70 ± 0.01
	Davies Reef	2013/14	1.58 ± 0.36	1.53 ± 0.02	2.43 ± 0.10
	Heron Island	2013/14	1.43 ± 0.36	1.67 ± 0.03	2.40 ± 0.12

#### *Pocillopora damicornis*

Average linear extension of *P*. *damicornis* was higher at Lizard Island (1.04 ± 0.09 cm 6-month^−1^), compared to Davies/Trunk Reefs (0.91 ± 0.11 cm 6-month^−1^) and Heron Island (0.69 ± 0.09 cm 6-month^−1^). There were significant differences in linear extension between Lizard and Heron Island (Fig. 3D; lme, t = −3.270, p = 0.011; Supplementary Table S2), but intermediate levels recorded at Davies/Trunk Reef were not significantly different to those recorded at Lizard Island or Heron Island (lme, t = −2.17, p = 0.062). Significant seasonal differences in growth of *P*. *damicornis* was apparent at Lizard and Davies/Trunk (Nested ANOVA p < 0.05), but not Heron (Nested ANOVA, p > 0.05, Supplementary Table S3). Skeletal bulk density of *P*. *damicornis* was not significantly different across the latitudinal gradient of the Great Barrier Reef with an average density of 1.21 ± 0.02 g cm^−3^ (Fig. 3E; lme p > 0.05, Supplementary Table S3). There was also no variation in skeletal density with respect to season (Nested ANOVA p > 0.05, Supplementary Table S3). Calcification rates per 6-months were greatest at Lizard Island (1.10 ± 0.06 g cm^−2^), significantly greater than Davies/Trunk (1.07 ± 0.07 g cm^−2^) and Heron Island (0.84 ± 0.06 g cm^−2^) (Fig. 3F; lme p < 0.05, Supplementary Table S2). Along the GBR, there was no significant variation in calcification between the 2013–14 summer and 2014 winter sampling periods (Nested ANOVA, p > 0.05, Supplementary Table S3). Calculated annual rates of linear extension, density and calcification are provided in Table 1.

#### *Isopora palifera*

Average linear extension across all locations for *I*. *palifera* was 1.49 ± 0.03 cm yr^−1^. Linear extension was greatest at Lizard Island (1.64 ± 0.06 cm yr^−1^) but was not significantly different to growth rates recorded at Davies Reef (1.51 ± 0.06 cm yr^−1^) or Heron Island (1.33 ± 0.06 cm yr^−1^) (lme, p > 0.05; Supplementary Table S2, Fig. 3G). In contrast, annual skeletal density was greatest at Heron Island (1.33 g cm^−3^) compared to the Lizard Island and Davies Reef (Fig. 3H), but did not significantly vary between reefs (lme, p > 0.05, Supplementary Table S2). Annual calcification rates were only 8% greater at Lizard Island (2.69 ± 0.22 cm yr^−1^) compared to Davies Reef and Heron Island (Fig. 3I) and were not significantly different (lme, p > 0.05; Supplementary Table S2).

### Environmental variables along the Great Barrier Reef and their influence on coral growth

#### Sea surface temperature

**Figure 4:**
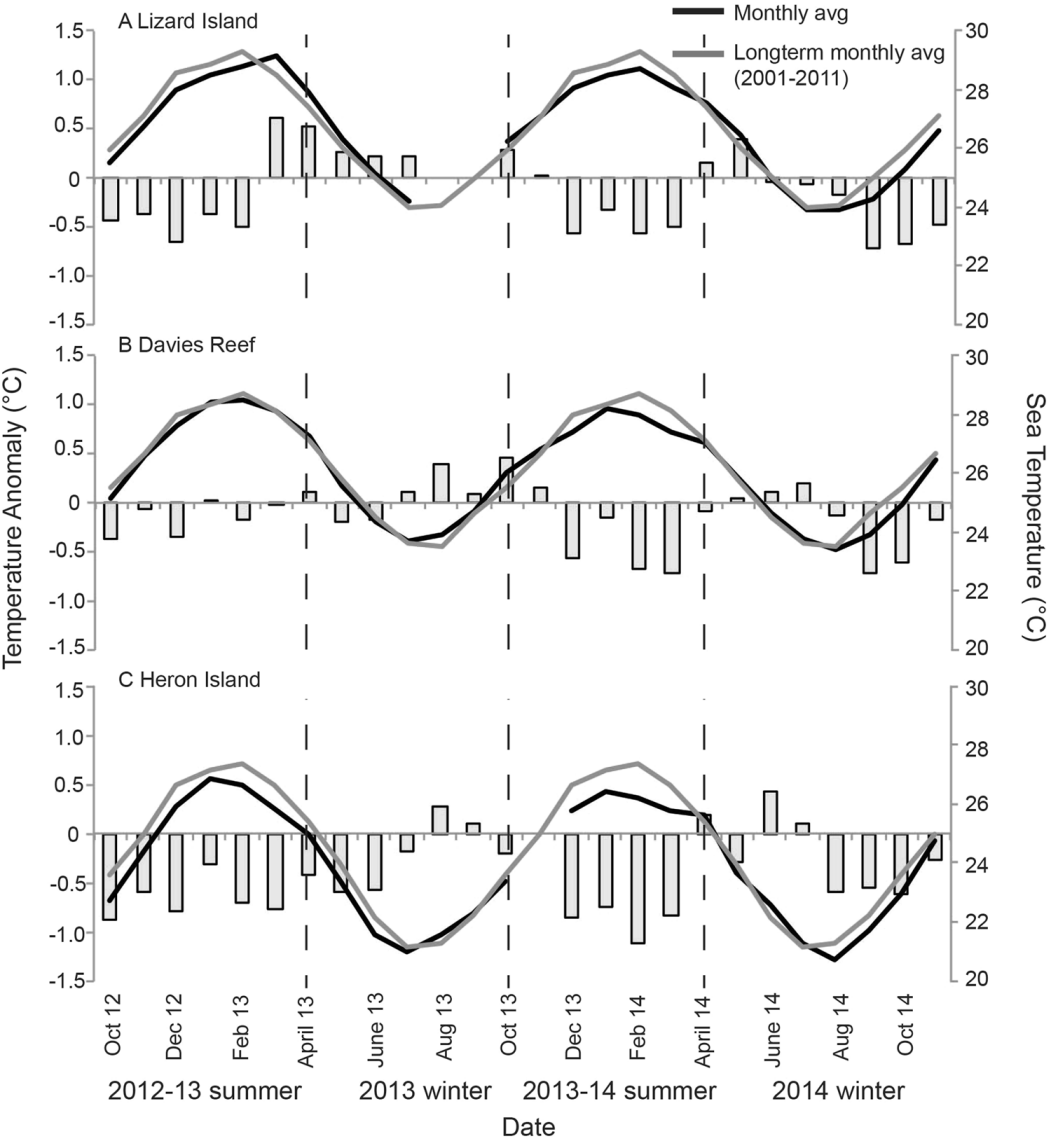
Seasonal temperature profiles for (**A**) Lizard Island, (**B**) Davies Reef and (**C**) Heron Island during the course of the study (October 2012–Nov 2014). The average monthly sea temperature (°C) and the long-term monthly average sea temperature determined from 2001–2011 are plotted. The temperature anomaly between the study period monthly average and the long-term monthly average is displayed in bars.

There was a significant variation in both average annual SST (Two-way ANOVA, F_2/76_ = 196, p = 0.005) and monthly average SST (Two-way ANOVA, F_24/76_ = 36, p = 0.027) among reefs. Throughout the course of the study (Oct 2012–Nov 2014), the average SST at Lizard Island was 26.8 °C (ranging from 23.2 to 30.6 °C), compared to 26.1 °C (22.5–30.3 °C) at Davies Reef and 24.1 °C (17.4–28.6 °C) at Heron Island. Summer temperatures in both 2012–13 and 2013–14 were lower than the long-term monthly averages for all locations, ranging from ~0.5 °C cooler than expected for Lizard Island to 1.0 °C cooler than expected for Heron Island (Fig. 4). When comparing winter temperature anomalies among locations, the lower latitude sites of Lizard Island and Davies Reef had a greater preponderance of positive temperature anomalies in the winter months, when compared to Heron Island.

**Figure 5:**
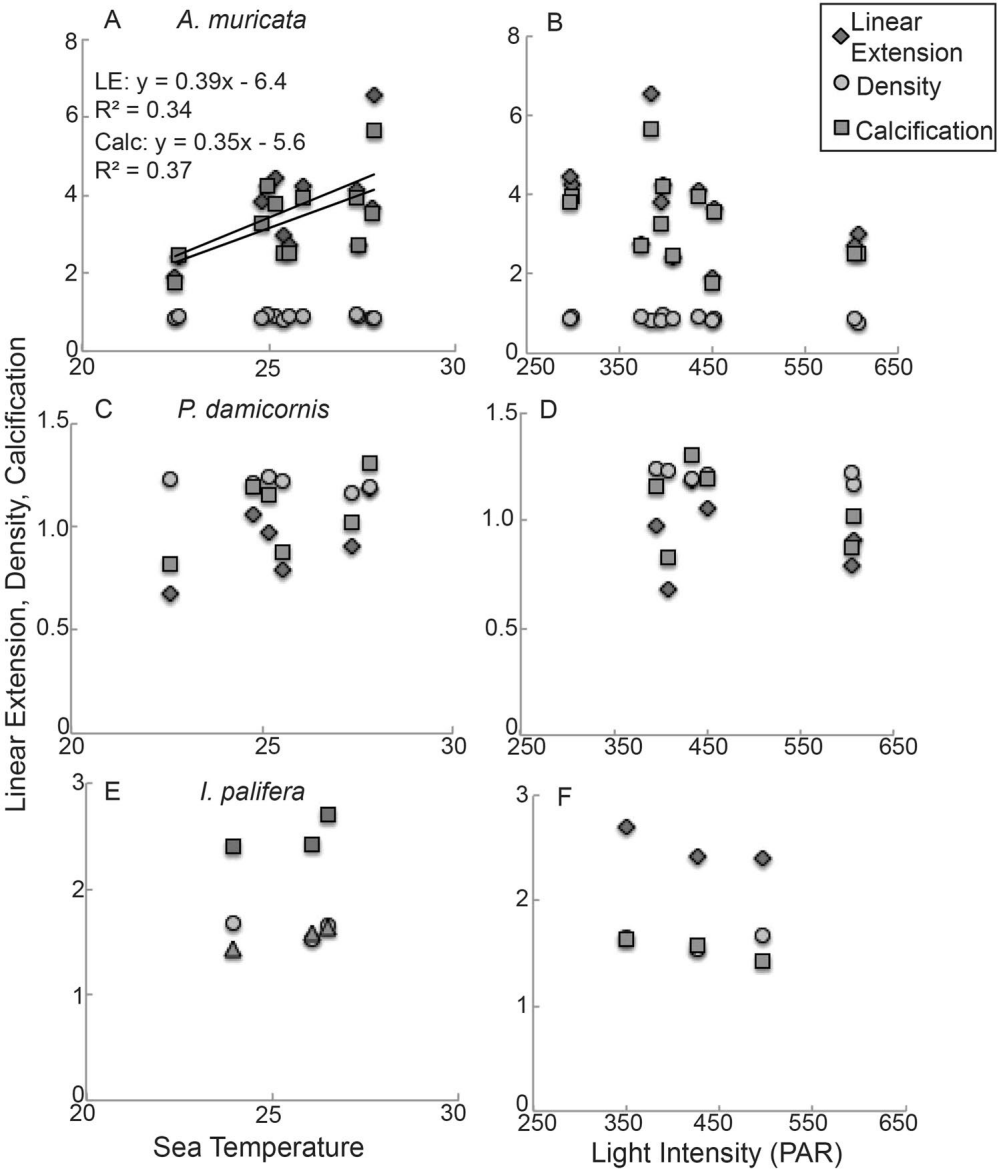
The relationship of summer and winter linear extension (cm 6-month^−1^), density (g cm^−3^) and calcification (g cm^−2^ 6-month^−1^) of *Acropora muricata* (**A**,**B**) *Pocillopora damicornis* (**C**,**D**), *Isopora palifera* (**E**,**F**) from 2012–2014 against average seasonal (6-month) sea surface temperature (°C) and light intensity (PAR) for all reef locations (Lizard, Davies, Heron) and sampling periods for each species. Line of best fit delineates a significant relationship.

For *A*. *muricata*, linear extension (cm 6-month^−1^) and calcification (g cm^−2^ 6-month^−1^) were significantly related to SST (linear extension: lm, F_1/10_ = 5.058, p = 0.048, R^2^ = 0.34; calcification: lm, F_1/10_ = 5.774, p = 0.037, R^2^ = 0.37, Supplementary Table S4, Fig. 5A,B); the linear extension and calcification increased 0.39 cm 6-month^−1^ and 0.35 g cm^−2^ 6-month^−1^, respectively, for each 1 °C of SST. While linear extension and calcification of *P*. *damicornis* did increase with SST (Fig. 5C,D), the relationship was not significant (lm, p > 0.05; Supplementary Table S4).

Investigating the historic SST using the long-term dataset at each reef, the annual average at Lizard Island from 1965–2000 was 25.83 ± 0.07 (Supplementary Fig. S3). The average long-term SST values from 1965–2000 compared to the study period (2012–2014) were 0.12 °C higher (26.02 ± 0.07 °C) but did not significantly differ (t-test, t = 0.801, df = 37, p = 0.428; Supplementary Table S5). Comparable SST was observed at Davies Reef and Heron Island when comparing the average long-term SST values from 1965–2000 to the study period (t-test: Davies t = 0.299, df = 37, p = 0.767, Heron t = 0.270, df = 37, p = 0.789; Supplementary Table S5). There was a stark contrast when comparing the rate of increase of the annual average SST during that time; at Lizard Island the SST rate of increase (0.11 °C per decade) was 35% greater compared to Davies Reef (0.07 °C per decade) and 47% greater compared to Heron Island (0.06 °C per decade). Frequency of summer extremes, which likely result in lower than optimal growth rate, were examined using the Degree Heating Week (DHW)^32^. The frequency of recorded DHW > 0 °C-week events was markedly greater at the two lowest latitude sites in Lizard Island (35/50 years, Supplementary Fig. S3a) and Davies Reef (31/50 years, Supplementary Fig. S3b) compared to Heron Island (23/50 years, Supplementary Fig. S3c).

#### Light

Comparing average monthly light intensity (PAR) among sectors, there was a significant variation among sectors (two-way ANOVA, F_2/30_ = 20.68, p < 0.001) and month (two-way ANOVA, F_15/30_ = 2.99, p = 0.005, Supplementary Fig. S4). Average monthly PAR (μmol s^−1^ m^−2^) during the study was greatest at Heron Island (539 ± 27 μmol s^−1^ m^−2^), compared to Lizard Island (375 ± 30 μmol s^−1^ m^−2^) and Davies Reef (371 ± 22 μmol s^−1^ m^−2^). When examining the relationship between calcification, density and linear extension rates of *A*. *muricata* and *P*. *damicornis* with light intensity (PAR), for all growth variables, there was no significant relationship (lm, p > 0.05; Supplementary Table S4, Fig. 5).

#### Aragonite saturation

Aragonite saturation determined over a 9-day period (23 Jan–1 Feb 2014) at Lizard Island averaged 3.3 ± 0.2 (Table 2) but ranged from 2.6–3.7 (Supplementary Fig. S5). Comparable estimates of aragonite saturation for Davies Reef (17–27 January, 2012) averaged 3.7 ± 0.2^33^ and at Heron Island (8–18 March, 2012) averaged 3.3 ± 0.03^34^. Diel patterns in aragonite saturation determined for Lizard Island (Supplementary Fig. S5) follow the similar trends as observed for Davies Reef^33^ and Heron Island^34^. At Lizard Island, the pCO_2_ and total inorganic carbon (C_T_) were on average highest just before dawn (Supplementary Fig. S6), but there were two days when pCO_2_ at 2:00 am was greater than 550 ppm (28/01–29/01), coinciding with a period of extensive rainfall at Lizard Island (Supplementary Fig. S7). pCO_2_ levels were highly variable but followed a pattern of increasing throughout the day and were lowest around dusk. Diel patterns in pH and aragonite saturation (Ω_arag_) were inverse to those of pCO_2_ and C_T_ but with the highest values at dusk and lowest at dawn (Supplementary Fig. S6). All measured and calculated physical and chemical parameters are presented in Table 2, along with those previously determined for Davies Reef and Heron Island.

**Table 2:** Comparison of within-reef carbonate chemistry at Lizard Island (northern), Davies Reef (central) and Heron Island (southern) of the Great Barrier Reef.

Location	T^*^ °C	S^*^	A_T_^*^ μmol kg^−1^	C_T_^*^ μmol kg^−1^	pH_seawater_	*p*CO_2_ μatm	Ω_arag_	CO_3_^2−^
Lizard Island (mean)	28.5 ± 0.3	34.6 ± 0.3	2230 ± 24	1941 ± 26	7.99 ± 0.03	449 ± 46	3.3 ± 0.2	206 ± 14
range	27.7–29.4	34.0–35.2	2165–2263	1874–2001	7.86–8.06	369–644	2.6–3.7	369–644
Davies Reef (mean)	28.5 ± 0.2	35.0 ± 0.1	2276 ± 16	1954 ± 25	8.03 ± 0.03	404 ± 40	3.7 ± 0.2	228 ± 13 227
range	28.1–28.9	34.9–35.1	2213–2304	1878–2018	7.92–8.10	325–542	2.9–4.1	181–253
Heron Island (mean)	26.6 ± 0.1	35.4 ± 0.0	2258 ± 2	1969 ± 4	7.99 ± 0.00	449 ± 6	3.3 ± 0.0	205 ± 2
range	24.2–30.4	35.2–35.5	2176–2307	1834–2059	7.83–8.14	281–669	2.3–4.2	144–261

Values for Davies Reef and Heron Island determined by Albright *et al*.^33, 34^, respectively. Averages and ranges of measured* and calculated physical and chemical parameters given. Carbonate chemistry for Lizard Island was determined in this study in January 2014 on the back reef inside the Lizard Island lagoon (Fig. S1). Carbonate chemistry was determined on the back reef flat at Davies Reef in the summer January 2012. In Heron Island, values are for the combined sampling on the reef flat and crest in March 2012. T = temperature, S = salinity, A_T_ = Total alkalinity, C_T_ = Carbon total, *p*CO_2_ = partial pressure of carbon dioxide, Ω_arag_ = Aragonite saturation, CO_3_^2−^ = carbonate ion.

### Meta-analysis of *Acropora muricata* and *Pocillopora damicornis* growth rates

**Figure 6:**
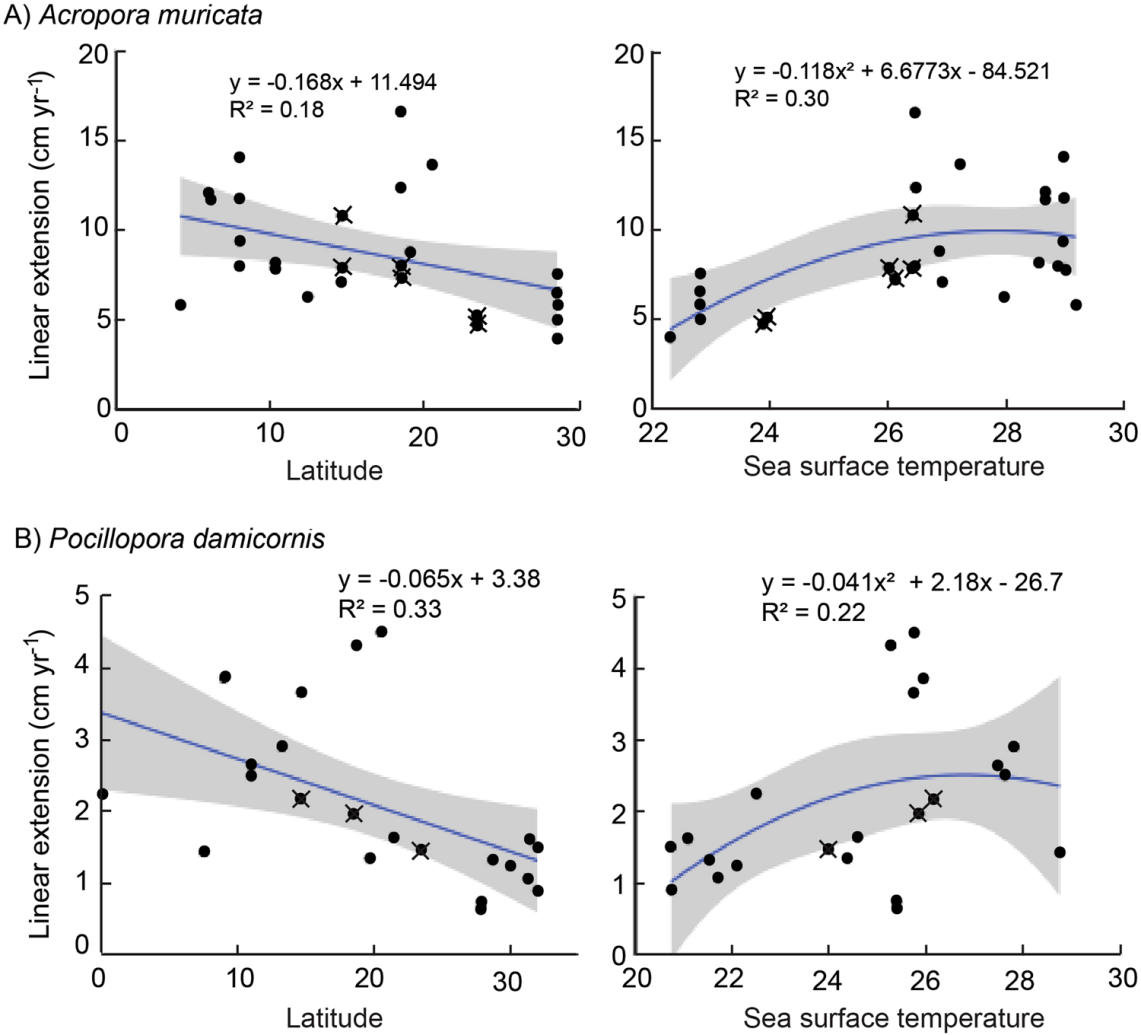
Variation in linear extension rates (cm yr^−1^) of (**A**) *A*. *muricata* and (**B**) *P*. *damicornis* with latitude and sea surface temperature (°C). Data points arisen from this study are dots with crosses. Values for data points of *A*. *muricata* are in Supplementary Table S7 and *P*. *damicornis* in Supplementary Table S8. Shaded area represents 95% confidence intervals.

From the meta-analysis on linear extension rates for *A*. *muricata* (Supplementary Table S7), linear extension rates of *A*. *muricata* decreased with increasing latitude (lm, F_1/25_ = 5.331, p = 0.030, R^2^ = 0.18). As well, linear extension rates varied among locations in approximate accordance with spatial variation in annual average SST (lm, F_2/24_ = 5.156, p = 0.014, R^2^ = 0.30, Fig. 6A).

*Pocillopora damicornis* is a ubiquitous species with many estimates of growth rates published in the literature (Supplementary Table S8). However, there is a significant difference in rates of linear extension in the Indo west-Pacific (2.0 cm ± 0.3) being half of those reported for the Eastern Tropical Pacific (4.2 cm ± 0.3) (one-way ANOVA, F_1/33_ = 27.97, p < 0.001). Therefore, the relationship between SST and latitude focused solely on data from the area of interest, the Indo west-Pacific. There was a significant negative relationship of decreased linear extension with increasing latitude (Fig. 6B, F_1/20_ = 9.83, p = 0.005, R^2^ = 0.33). Linear extension increased with SST to an optimal value at 26.7 °C (Fig. 6B), however, the relationship was not significant (lm, F_2/19_ = 2.706, p = 0.093, R^2^ = 0.22).

## Discussion

This study showed that growth rates of three common coral species (*A*. *muricata*, *P*. *damicornis* and *I*. *palifera*) were highest at Lizard Island, in the northern GBR (14.7 °S), and declined at higher latitude. Spatial variation in coral growth was largely consistent with variation in observed temperatures; average annual SST at the two most northern reefs were similar (26.8 °C and 26.1 °C, at Lizard Island and Davies Reef, respectively), however sea temperature at Heron Island was ~2 °C less (24.1 °C). Growth rates of *A*. *muricata* increased by 1.9 cm yr^−1^ for every 1 °C increase in average annual temperature across the three study locations. For *P*. *damicornis*, linear extension increased 0.3 cm yr^−1^ per 1 °C among locations. Despite contrasting morphologies, the rate of increase for *P*. *damicornis* was the same as that determined for massive morphologies of *Porites* (0.31 cm yr^−1^ per 1 °C) on the GBR^15^. Growth rates of *Isopora* increased by 0.11 cm yr^−1^ per 1 °C among the three study locations.

In addition to temperature, another key environmental driver of coral reef calcification is aragonite saturation^13^. While we see large gradients in aragonite saturation in the open oceans^35^, within-reef saturation states are driven by complex interactions of photosynthesis/respiration, tides, benthic composition and factors that affect the biological activity such as temperature, light, salinity, and nutrients^33, 34, 36, 37^. Heavy rainfall and large changes in tide during deployment of the water sampler at Lizard Island likely contributed to a small reduction in aragonite saturation. However, the mean value determined in this study (Ω_arag_ = 3.3) is similar to that determined at Lizard Island in Oct–Nov 2008 and 2009 (Ω_arag_ = 3.65 and 3.45, respectively)^38^ The average for the central sector at Davies Reef was slightly higher (Ω_arag_ = 3.7)^33^ than the other sectors and may reflect the variance that can be observed due to factors outlined above. Importantly, variation among locations in growth rates of the three corals species considered in this study (*A*. *muricata*, *P*. *damicornis* and *I*. *palifera*) was more consistent with differences in local temperature, rather than light or aragonite saturation.

Mean within-reef aragonite saturation states were comparable (Table 2: Ω_arag_ = 3.3) at Lizard Island and Heron Island, despite 8.76° latitudinal separation and 2 °C difference in SST. It seems despite lower temperatures and concomitant greater solubility of calcium carbonate at Heron Island, the benthic community at Heron Island can buffer the reef carbonate saturation state^39^ and maintain an average aragonite similar to the lower latitude site of Lizard Island. However the variability in available carbonate ions (Ω_arag_) at Heron Island (range = 2.1, Ω_arag_ 2.3–4.2) is almost double that of Lizard Island (range = 1.1, 2.6–3.7). The reef flat of Heron Island is characterised by a greater seawater residency time that can result in large fluctuations in carbonate chemistry^37, 40^. Therefore, the locally adapted corals of Heron Island at the southern GBR may be able to withstand changes in seawater chemistry, as they are naturally subjected to large intra-annual variation in temperature and pH^34, 40^. However, periods of anomalous temperature and pH exposure do not coincide at Heron Island; the greatest diel temperature range was recorded in austral spring (October) and largest diel variability in pH occurred in the fall (June)^40^. It is likely that the effects of increasing temperature on coral growth will be exacerbated with declining pH^41^ but temperature-induced increases in coral metabolism^42^ may counteract any negative response such that the synergistic affects remain unclear. Understanding how these stressors synergistically affect coral growth and survival will be imperative for predicting persistence of the reef community.

Meta-analyses of large-scale variation in growth (linear extension) for both *A*. *muricata* and *P*. *damicornis* revealed generally increasing linear extension rates with decreasing latitude, as shown previously for massive *Porites* ^15^. While temperature may be just one of several environmental parameters that vary with latitude, and thereby account for observed differences in growth^12^, it is striking how these spatial patterns correspond to variation in local annual mean temperatures. Notably however, the best model to describe the relationship between linear extension rates of both *A*. *muricata* and *P*. *damicornis* with SST is a saturating relationship, showing that growth is largely invariant to changes in annual mean temperature between 26 and 30 °C (Fig. 6).

A meta-analyses was not completed on *Isopora palifera* as this is the first study to publish growth characteristics on this species. The only other record of linear extension for *Isopora sp*. (*I*. *cuneata*) was at a subtropical location in Australia, Lord Howe Island, that averaged 1.96 ± 0.07 cm yr^−1^ from 2010–11^43^. This is a greater growth rate than reported for the genus in this study on the GBR (1.43–1.64 cm yr^−1^) but comparison between the two studies is flawed as the species and methodologies between the two studies differed. The colonies from Lord Howe Island were not sliced to the dominant axis of growth and linear growth may have been overestimated; In this study, initial investigation revealed the linear extension determined externally from the stain line to column tip without sectioning was overestimated by an average of 0.35 ± 0.1 cm (mean ± SE, N = 13). As a result, in this study all columns of *I. palifera* were sectioned to determine linear extension.

The effects of increasing SST on coral growth will vary spatially, depending upon the local thermal history, the extent to which corals are locally adapted, and the extent to which growth is constrained by cool winter or warm summer temperatures^12^. At the low latitude site Lizard Island, the lowest growth rate of *A*. *muricata* was in the 2012–13 summer (Fig. 3A), a summer that started to be on average cooler, but by March–April was ~0.5 °C greater than the 10-year long-term average (Fig. 4A) which may have caused sublethal effects of reduced growth. The positive temperature anomalies continued into the 2013 winter (Fig. 4A) and may have contributed to growth rates of *A*. *muricata* being sustained over the winter months. At Lizard Island, *A*. *muricata* growth during the 2013–2014 summer was 30% greater than all other sampling periods. During this summer, that majority of these months were 0.5 °C less than the long-term average suggesting temperatures at Lizard Island are already past their thermal optimum in the summer and reductions of 0.5 °C (to ~28.5 °C) from what is often experienced would be more favorable for growth. This trend of constrained coral growth in summer months has also been reported in Western Australia^44^ and in the Persian Gulf^45^. At the higher latitude site of Heron Island, positive temperature anomalies in the winter months may be leading to higher growth rates, unrestricted by cooler temperatures. Similarly, *P*. *damicornis* did not display expected seasonal trends in growth rates (Fig. 3B). At all locations, the 2013–14 summer had negative temperature anomalies compared to the previous 10-years (Fig. 4). Moreover, the beginning of the 2014 winter months was unusually warm, which may have allowed *P*. *damicornis* growth rates to remain similar to the previous summer. Thus, temporal trends in growth rates of branching species on the Great Barrier Reef exhibited high seasonal variability with no clear trend with respect to temperature; unusually warm winters at Heron Island and unusually cool summers at Lizard Island were associated with the highest growth rates obscuring expected seasonal trends.

Future growth rates of corals are likely to be increasingly constrained by temperatures exceeding the optimal for coral growth and survivorship^46^ and it is evident that lower latitude locations on the GBR are already experiencing an increased severity of thermal stress (Supplementary Fig. S3). Increasing temperature is the biggest threat to corals due to the predicted increase in occurrences of coral bleaching events^47^. The Great Barrier Reef in 2016 had just experienced its third mass bleaching event with the two previous bleaching events in 1998 and 2002^17^. There was spatial variability between the events, with 1998 and 2002 events largely impacting the central GBR and the 2016 event impacting the lower latitudes of the GBR^17^. With repeated bleaching events, these sites may experience shifts in the coral composition to those more thermo-tolerant species, leading to declines in structural complexity which would have important consequences on fishes and mobile invertebrates associated with live coral habitats^2, 30, 48^.

High latitude sites such as the southern GBR may initially serve as refugia from thermal stress. However, even high latitude sites can be affected by thermal stress or declining saturation state leading to reductions in growth rates^43^. The relative benefits of increasing temperature versus constraints imposed by declines in aragonite will play a dominant role in determining the fate of coral in the future^46^. Heron Island already exhibits reduced rates of coral calcification (50% less for staghorn *A*. *muricata*, 30% less for P. *damicornis* and 8% less for *I*. *palifera* (Fig. 3)) compared to those at Lizard Island, likely driven by considerably lower annual average sea temperature. Therefore, increases in temperature may have a positive effect on coral growth at the southern GBR, but there is limited understanding of the constraints imposed by ocean acidification on coral accretion at these high latitude locations. Overall, declines in aragonite saturation are expected to be less influential on calcification than rising sea temperature^49, 50^ throughout this century.

In conclusion, growth rates of three dominant coral species varied spatially along the GBR, largely in conjunction with differences in average annual temperatures. Based on limited temporal sampling of carbonate chemistry, within-reef aragonite saturation was comparable among these locations, suggesting that reef ecosystems do have considerable capacity to buffer latitudinal gradients in oceanic aragonite saturation. There was also no consistent trend in variation in solar radiation among reefs relative to observed variation in growth rates, though this may impose increased constraints on coral growth at higher latitudes^51^. Linear extension rates at Heron Island, where annual SST was on average 2 °C lower, were 20%, 33%, and 34% less for *I*. *palifera*, *P*. *damicornis* and *A*. *muricata*, respectively, compared to Lizard Island. Ocean warming may therefore, lead to moderate increases in growth rates of corals at southern locations (see also Pratchett *et al*.^12^), whereas increasing temperatures may increasingly constrain coral growth (especially during summer) at lower latitudes. Continued monitoring of coral growth rates in combination with environmental conditions, such as temperature stress and within reef carbonate chemistry, will be essential to better appreciate likely impacts of climate change on corals and reef ecosystems.

## Methods

### Coral species

Coral growth (specifically, linear extension, density, calcification) was quantified for three coral species (*Acropora muricata* (cf. *A*. *formosa*), *Pocillopora damicornis*, and *Isopora palifera*) that were common and abundant at widely separated locations along the length of the GBR. *Acropora muricata* is a staghorn coral that often dominates in shallow water lagoons, forming large mono-specific thickets. *P*. *damicornis* is an abundant, ubiquitous species and the recent amendment of the *Pocillopora* complex taxonomy^52^ was used to aid identification of *P*. *damicornis*. *I*. *palifera* is abundant in high wave energy areas forming submassive clumps that contain columns and ridges^53^.

### Study sites

This study was conducted at three distinct locations, spread along 1,187 km of Australia’s Great Barrier Reef (Fig. 1). The northernmost location was Lizard Island (14.7°S), followed by Davies Reef (18.8°S) and Trunk Reef (18.4°S), and finally Heron Island (23.4°S) in the south. At each of the three locations, coral growth was documented at 2 sites for *A*. *muricata* and *P*. *damicornis*, but only 1 site for *I*. *palifera*. Study sites were specifically selected to provide comparable habitat and depth (5 m) at each reef, while also selecting areas with high abundance of each of the specific study species. In the central sector, *P*. *damicornis* was poorly represented at the first reef visited (Davies Reef) and therefore, additional sampling was undertaken at another nearby reef, Trunk Reef (Fig. 1I).

### Coral growth

To quantify growth rates for each coral species, replicate colonies and/or branches were stained *in situ* using Alizarin Red^54^. As calcification takes place, the dye gets incorporated into the skeleton producing a permanent reference against which to measure all subsequent skeletal growth. The concentration of Alizarin used was 12 mg L^−1^ and corals were exposed to the dye for 4 hours during the daylight hours of 900–1600^54^. Stained corals were marked with cattle tags attached to the colony base, away from the growing tips to minimise disruption to growth. In total, 240 colonies of *A*. *muricata*, 120 colonies of *P*. *damicornis* and 30 colonies of *I*. *palifera* were stained. Once collected, branches and colonies were placed in 10% sodium hypochlorite to remove the coral tissue and expose the skeleton (Fig. 2).

#### *Acropora muricata*

The sampling regime for *A*. *muricata* encompassed four 6-month periods at each reef: summer 2012–2013 (Oct/Nov 2012–Mar/Apr 2013), winter 2013 (Apr 2013–Oct 2013), summer 2013–2014 (Oct/Nov 2013-Mar/Apr 2014), winter 2014 (Apr/May 2014–Oct-Nov 2014). Individual branches were enclosed in a 1 L plastic bag *in situ* and secured with an elastic band. Alizarin red was injected with a needle under the elastic band into the bag. Ten colonies of *A*. *muricata*, with three branches per colony were stained at each site, totalling 20 colonies and 60 branches per reef (60 colonies/180 branches total) in October 2012. In April 2013, the stained colonies were collected and another set stained. This cycle of staining continued for two-years totalling 720 branches.

Growth measurements were determined for each branch of *A*. *muricata*. Linear extension (cm) was measured with Vernier calipers, recording the minimum distance from the stain line to tip of the apical polyp. Branches that had died prior to final collection were excluded from analysis. In addition, 0.02% (16/720) of branches had switched to zooxanthellae free (non-growing) tips through the course of the study that can result from interior conditions of the colony being less suitable for growth or branches growing too closely together^55^ and were excluded from analysis. To determine bulk skeletal density of the branching species *A*. *muricata*, corals were cut along the stain line with a geological saw using a 2 mm saw blade. The dry weight (g) of the cut branch tips was recorded. Branches were dipped in paraffin wax and the total enclosed volume was determined using water displacement technique^56^. Skeletal bulk density (g cm^−3^) was then determined by dividing the dry weight by the enclosed volume. Calcification (g cm^−2^) rates were calculated by multiplying the linear extension by the density^15^. However, these calcification rates are only of the apical newly grown branch and are not a measure of whole colony calcification.

#### *Pocillopora damicornis*

The sampling regime for *P*. *damicornis* encompassed one year, separated into two 6-month periods at each reef: summer 2013–2014 (Oct/Nov 2013–Mar/Apr 2014) and winter 2014 (Apr/May 2014–Oct/Nov 2014). 20 colonies per location (10 at each site) were stained. Colonies (max 20 cm diameter) were removed from the substrate and put into a 70 L clear plastic bag *in situ* with Alizarin dye released into the bag. For *P*. *damicornis*, the number of coral branches measured with callipers ranged from 10–20 (n = 10 for corals 8–15 cm in diameter, n = 20 for corals 15–20 cm in diameter). Branch measurements for *P*. *damicornis* were randomly sampled along the longest axis of growth (often the most upward projecting). Measured branches were sectioned with a saw and the bulk density and calcification was determined as described for *A*. *muricata*. In addition, areas of partial mortality due to algal overgrowth or smothering due to sediment were excluded. Calculated rates of density and calcification were averaged among branches and determined at the colony level.

#### *Isopora palifera*

Growth rates of *I*. *palifera* were determined for an entire year (Oct/Nov 2013–Oct/Nov 2014) at one site per location (Lizard Island, Davies Reef, Heron Island). At each location, 10 colonies were stained (30 colonies total) with 2–3 columns per colony. Columns of *I*. *palifer*a were enclosed in a 4 L clear plastic bag, sealed at the base with an elastic band and the dye injected under the elastic band with a needle. To determine the linear extension of *I*. *palifera*, stained coral columns were sliced at 4.4 mm thickness until the maximum vertical axis of growth was determined and then photographed with a reference scale. Linear extensions were determined for each column by taking three measurements along the main vertical growth axis from the stain line to the end of the column on the digital image in Image J Fiji^57^. Density and calcification rates of *I*. *palifera* were determined from the digitised images of x-radiography adapted from Carricart-Ganivet & Barnes^58^ and detailed methodologies can be found in Supplementary Materials.

### Statistical Analysis

To assess spatial variation (among locations) in linear extension, calcification, and density of corals, the package nlme^59^ was used to fit linear mixed-effects (lme) models using the statistical program R^60^. Separate analyses were conducted for each coral species (*Acropora mucicata*, *Pocillopora damicornis*, *Isopora palifera*). For all models the fixed effect was location and as a random effect sampling period which was nested in site and then colony. Model selection was informed by Akaike information criterion (AIC) which measures the relatively quality of the model for the data set. The best models were selected based on lowest AIC, then fit by restricted maximum likelihood (Supplementary Table S1). Model assumptions, including normality of errors and homogeneity of variances, were evaluated graphically. To correct for heteroscedasticity and non-normality, a square-root transformation to the models was used (Supplementary Table S1). For each species, one-way analysis of variance (ANOVA) was used post hoc to investigate temporal variation in growth parameters within each location.

Annual rates of linear extension (cm yr^−1^), density (g cm^−3^) and calcification (g cm^−2^ yr^−1^) were determined averaging the 6-month means (x) for *A*. *muricata* and *P*. *damicornis* and standardizing for variation in sample size (N) between study periods using equation 1:

1 $${{\rm{X}}}_{6-{month}}=\frac{\sum ({\rm{x}}\ast \,N)}{{\rm{\Sigma }}N},$$

which was then converted to an annual rate.

### Environmental Parameters

#### Sea surface temperature and light

Sea surface temperature (SST) and light (PAR) throughout the study was determined from the Integrated Marine Observing System (IMOS, http://www.aims.gov.au/docs/data/data.html, accessed March 6, 2015) and details of determining monthly averages found in Supplementary Materials. For each location, the long-term (2001–2011) average monthly SST was determined for comparison to the study period (2012–2014). Two-way ANOVA was used to determine significant variation between location SST/light and monthly time period. The relationship of *A*. *muricata* and *P*. *damicornis* density, linear extension and calcification rates were compared to the sampling period average SST and light for each reef and sampling period using linear regression (lm). As *I*. *palifera* was only investigated for one year at 3 sites, a statistical relationship cannot be reliably established due to limited sample size.

Historic sea surface temperature trends were determined from the Extended Reconstructed Sea Surface Temperature (ERSST) dataset (2° and monthly resolution; www1.ncdc.noaa.gov/pub/data/cmb/ersst/v3b/netcdf/, accessed August 2015). Satellite SST data of higher-resolution, both spatially (1/24°, ~4 km) and temporally (weekly), were also derived for the period 1985–2012 from the Pathfinder v5.2 night-only, 4 km-daily SST dataset (pathfinder.nodc.noaa.gov, accessed October 2013), with gaps filled following Heron *et al*.^61^. These data were recently used by Heron *et al*.^62^ to examine recent temperature trends and thermal stress patterns. However, as these data did not overlap the time periods of interest, 4 km-monthly composites were calculated and used to bias adjust the spatially and temporally lower resolution ERSST data to ensure relevance to the study. Trends in bias-adjusted ERSST were calculated annually from 1965 to 2014. In addition, accumulated thermal stress was calculated from ERSST using the Degree Heating Months (DHM) metric. Values were converted to Degree Heating Weeks (DHW^32^) for ease of comparison with established ecologically-relevant thresholds for significant bleaching and mortality of 4 and 8 °C-weeks^32^. At each reef, bias-adjusted ERSST during the course of the study (2012–2014) was compared to the long-term average (1965–2000) using a Student’s t-test.

#### Aragonite Saturation

Within-reef aragonite saturation had previously been determined at Davies Reef^33^ and Heron Island^34^ but was yet to be quantified using the same methodologies in the northern sector at Lizard Island. The carbonate chemistry was determined in the summer for 9 days from Jan 23 to Feb 1, 2014, inside the lagoon on the protected back reef flat, following Albright *et al*.^33, 34^. Detailed description of the methodologies can be found in Supplementary Materials.

### Meta-analyses of coral growth

Linear extension is the metric most often reported in the literature for quantifying growth of branching corals^12^. To relate measurements of linear extension from along the GBR to other measure ments taken from further afield, separate meta-analyses was performed for *A*. *muricata* and *P*. *damicornis*, specifically testing for variation in linear extension with local average SST during the studies, as well as latitude. Only one other study had reported growth rates on *Isopora* at a subtropical location in Australia^43^ and therefore this species does not have enough data for a meta-analysis. A total of 13 and 16 studies that measured linear extension of *A*. *muricata* and *P*. *damicornis*, respectively, were used. Where applicable, if additional experimental conditions were imposed in the study, we used only growth estimates from “control” corals. SST was quantified (ERSST bias-adjusted using 4-km satellite values, as described above) for the dates of each study where provided; when sampling dates were not included, the year prior to publication was used. Study depths ranged from 0.5–15 m for *A*. *muricata* and 2–7 m for *P*. *damicornis* but depth was not reported in all the studies and could not be included in the analysis (Supplementary Tables S7, S8). However, the main question was to determine the relationship of linear extension across large temperature (annual average 21–29 °C) and latitudinal (0–30°) scales. Linear regression (lm) was used to investigate the fixed effect of linear extension on the random effects of average SST and latitude. For both the *A*. *muricata* and *P*. *damicornis* data set, the models with latitude had a linear model to explain the relationship best, whereas, for SST a polynomial function provided a better relationship (based on comparison of R^2^). For *P*. *damicornis*, the relationship of linear extension with latitude required a square root transformation to normalize the residuals.

Statistical analysis was completed in R^60^. All linear models were tested graphically to meet the assumptions of linear models (homogeneity of variance, linearity, independence).

### Data availability

The datasets generated during and/or analysed during the current study are available from the corresponding author on reasonable request.

## Electronic supplementary material

Supplementary Materials
