# Supplementary material for: Variation in growth rates of branching corals along Australia’s Great Barrier Reef
Source: Sci Rep. 2017 Jun 7;7:2920. doi: 10.1038/s41598-017-03085-1 (PMC5462780; doi:10.1038/s41598-017-03085-1)
Supplement: Supplementary file 1 — Supplementary Materials [file 41598_2017_3085_MOESM1_ESM.pdf]

Supplementary Materials

Variation in growth rates of branching corals along Australia's Great Barrier Reef

*Kristen D. Anderson[1,2], Neal. Cantin[2], Scott F. Heron[3,4,5], Chiara Pisapia[1], Morgan S. Pratchett[1]

[1] ARC Centre of Excellence for Coral Reef Studies, James Cook University, Townsville, QLD 4811, Australia

[2] Australian Institute of Marine Science, PMB 3, Townsville, Queensland 4810, Australia

[3] Coral Reef Watch, U.S. National Oceanic and Atmospheric Administration, College Park, MD 20740, USA

[4] Global Science & Technology, Inc., Greenbelt, MD 20770, USA

[5] Marine Geophysical Laboratory, Physics Department, College of Science, Technology and Engineering, James Cook University, Townsville, QLD 4811, Australia

Corresponding author: kristen.anderson3@jcu.edu.au

Supplementary Methods:

Aragonite Saturation at Lizard Island

An automated water sampler was deployed for 9 days from Jan 23, 2014 to Feb 1, 2014, between South Island and Palfrey Island (14°41'52.08 S, 145°26'58.05, Fig S1). The water sampler was deployed to document diurnal variability in carbonate chemistry. The approximate depth of the water sampler was 1.0 m but varied with the tides. The sampler pumped water at 2-hour intervals into pre-poisoned 250 mL borosilicate bottles (0.05% $HgCl_2$ to inhibit biological activity). Bottles were filled with reef water at a flow rate of ~2 mLs^{-1}. To avoid contamination of new samples, the sampler was programmed to flush the lines for 30s between filing. Filled bottles were retrieved every 12 hr and new, prepoisoned bottles were deployed.

Water samples were analysed at the Australian Institute for Marine Science (AIMS). Total alkalinity (A_T) and total dissolved inorganic carbon (C_T) were analysed using the VINTA 3C® (Versatile Instrument for Determination of Total dissolved inorganic carbon and Alkalinity, Marianda, Kiel, Germany) and a UIC CO_2 coulometer detector (UIC Inc., Joliet, USA). Accuracy was checked against certified seawater reference material. pCO_2, pH_T (total scale), and aragonite saturation state (Ω_{arag}) were calculated as a function of the measured salinity, temperature, A_T, and C_T using the program CO2SYS[66]; dissociation constants for carbonate and boric acid determined by Mehrbach et al. (1973) as refit by Dickson and Millero (1987), and the dissociation constant for boric acid determined by Dickson (1990), following Albright et al. (2013).

Benthic surveys were conducted on the reef flat between South and Palfrey Island at Lizard Island to characterize the surrounding community structure of the site. Five independent transects (20 m) were conducted perpendicular to the reef crest

on the back reef. Photographs were taken of 1 m^2 transects at 2 m intervals. Photos were analysed in Image J Fiji[56] for percentage cover of 1) live coral; (2) algae (macroalgae and turf); (3) crustose coralline algae (CCA); (4) $CaCO_3$ substrate (dead coral, rubble, sand); (5) and "other", including sponges, gorgonians, giant clams, etc.

Salinity (practical salinity units; psu) during deployment of the automated water sampler was determined from daily water samples. Samples were analysed at AIMS on a Portasal salinometer, which measure conductivity ratios and salinity.

Sea Surface Temperature and light

For SST at each reef, average, minimum and maximum daily and monthly SST from a sensor at 5-6 m depth was determined from Oct 2012-Nov 2014[64]. At the northern sector, Lizard Island SST was compiled from 3 sensors at 5-6.7 m to construct a 2-year consecutive assessment of SST (averaging data for multiple months). As the central GBR sector took place on two reefs, a t-test was performed on the average monthly SST to determine if there was significant variation between Davies Reef and the closest sensor deployed to Trunk Reef, Kelso Reef which is ~12 km apart). As there was no significant difference in temperature between the two central GBR sites ($t=0.126$, $df=33.9$, $p=0.815$), Davies Reef comprised a complete SST record and will be used for analysis. For light, the solar radiation sensor is an Under Water Quantum Sensor made by Licor that measures photosynthically active radiation (PAR) every 30 minutes[64] on the weather station relay poles. Daily averages were then determined and summed to a monthly value for analysis. To remove bias, analyses were performed on monthly data that were available for all sectors.

Determination of density and calcification for Isopora

In each X-ray exposure along with the coral, 6 standards made of compressed *Porites* skeleton powder, of increasing thickness (0.155-0.747 cm) and known

density, determined from weight (g) and volume (cm^3) were positioned alongside the coral skeleton. X-rays were taken with a Sectional CR High Resolution Digital Imaging System 3600+ (*i*CRco 2015) and converted for analysis in Image J Fiji[56]. A blank x-ray was subtracted to correct the background and the image was inverted for density extraction. The high quality of the scanner resulted in no further adjustments or corrections to the image. In each x-ray, the average optic density (OD) of each *Porites* standard was determined by creating a straight line of the natural log (lnOD) versus known aragonite density x step thickness. Linear fits to the lines provided correlation coefficients (R^2) better than 0.99. A standard (2.397 g cm^3) was used for quality control between consecutive x-rays producing a standard deviation of 1.6 % (0.04 g cm^3). A track 2.5 mm wide (roughly the size of a corallite) was run down the maximum vertical growth axis of each coral column and the optical density (grey-scale values of pixels ranging from 0-255) was sampled for each coral skeleton at 0.05 mm sampling interval. From the known linear relationship between the *Porites* aragonite standards, the OD (greyscale value) of each point was converted to skeletal density (g cm^{-3}). Annual density (g cm^{-3} yr^{-1}) along each column was determined from the known marked distance of annual growth from the stain line to the tip. Annual calcification (g cm^{-2}) was then calculated as the product of the annual linear extension from the stain line (cm) and annual density (g cm^{-3}) of new growth.

Supplementary Results:

Conditions during deployment of the automated water sampler at Lizard Island

Physical conditions at Lizard Island during measurements of seawater chemistry were conducive to high levels of water exchange, where wind gusts up to 32.5 knots (60 km/hr) with average wind speeds of 22.5 knots (40.62 km/hr)[64]. Moreover, the new moon was Jan 31, 2014 accompanied by the king tide causing water levels to range

from 0.02 m to 3.37 m. Average sea surface temperature during the 9 days was 28.5°C (ranging from 27.4 - 29.4)[64]. Benthic cover at Lizard Island was 17 % hard coral cover (Table S6). The greatest percentage of benthic cover (44.6 ± 7.4 %) consisted of $CaCO_3$ substrate due to a high proportion of sand patches and rubble on the reef flat. As well there is a large population of "other" substrate covering (29.9 ± 4.6 %), mainly gorgonians, covering the flat.

Supplementary References:

61. Lewis, E. & Wallace, D. W. R. Program Developed for CO2 System Calculations. Oakridge, Tennessee patent (1998).

62. Mehrbach, C. Measurement of the apparent dissociation constants of carbonic acid in seawater at atmospheric pressure. *Masters Thesis* (1973).

63. Dickson, A. G. & Millero, F. J. A comparison of the equilibrium constants for the dissociation of carbonic acid in seawater media. *Deep-Sea Research* **34**, 1733-1743 (1987).

64. Australian Institute of Marine Science (AIMS) Sea surface temperature data. AIMS Data Centre, accessed March 6, 2015. http://www.aims.gov.au/docs/data/data.html (2015).

Supplementary Figures

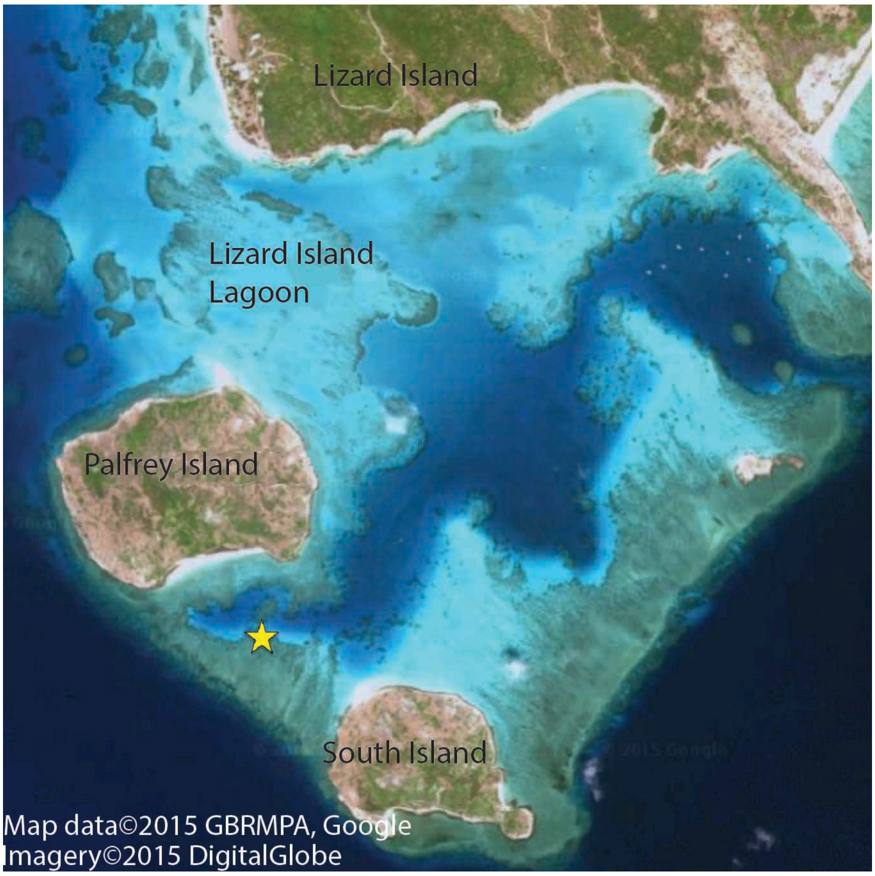

Figure S1. Location (star) of deployment of the automated water sampler in Lizard Island lagoon. Figure data provided by Imagery©2016 DigitalGlobe, Data SIO, NOAA, U.S. Navy, NGA, GEBCO, Map data ©2015 GBRMPA, Google 2015.

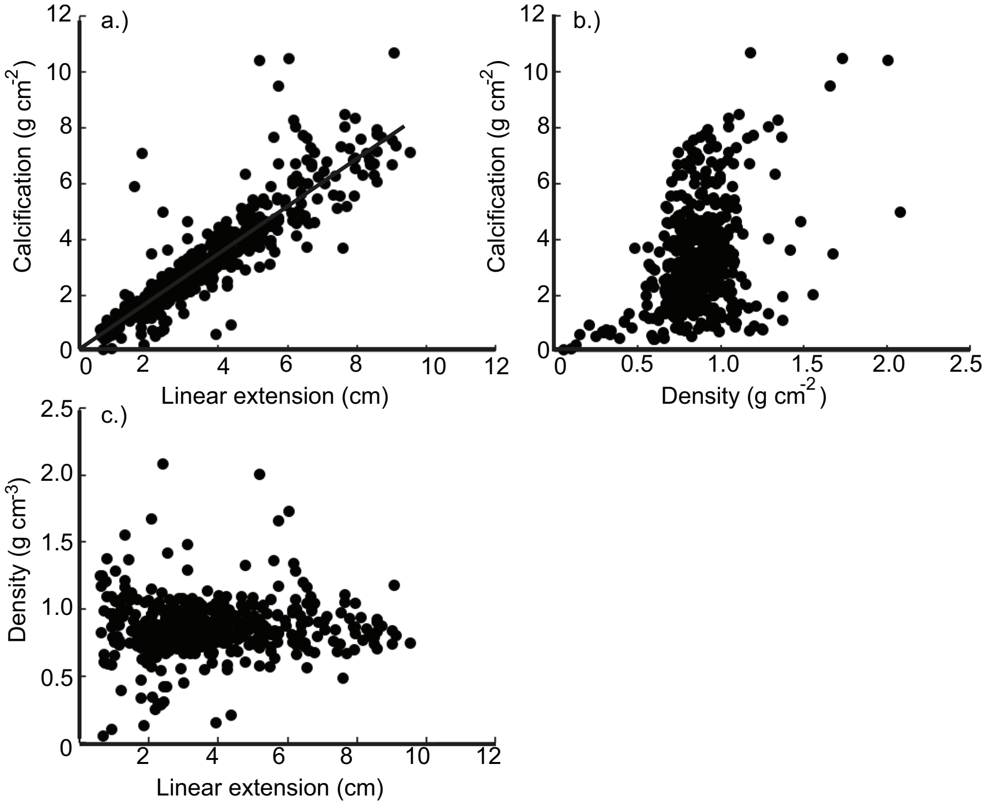

Figure S2. Relationship of calcification, linear extension and density of *Acropora muricata* branches during 6-month growing periods, collected along the Great Barrier Reef from Lizard Island, Davies Reef and Heron Island at 5 m depth.

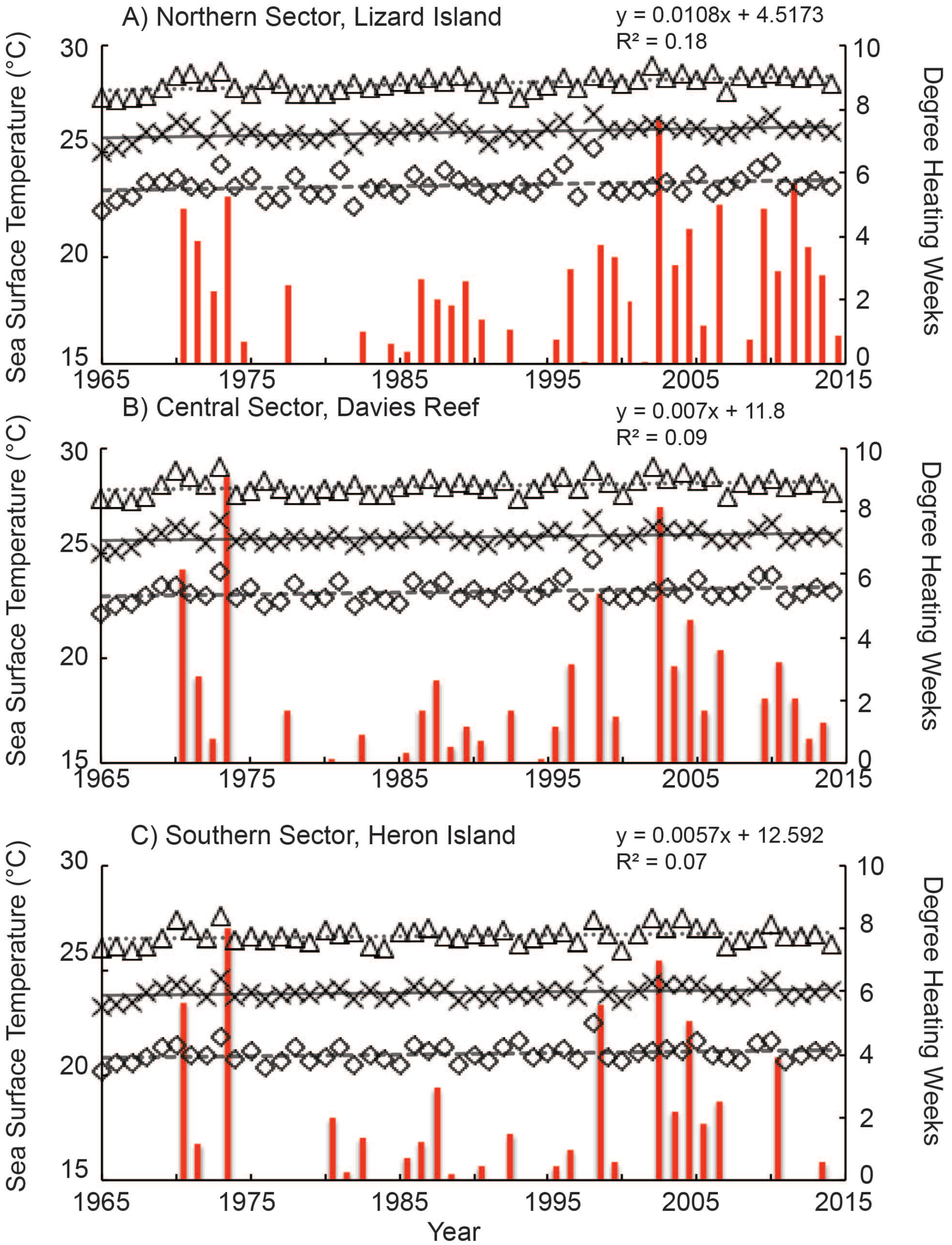

Figure S3. Relationship of average (X), maximum (Δ) and minimum (◊) sea surface temperature (°C) from 1965 to 2014 at the A) northern sector, Lizard Island, B) central sector, Davies Reef and C) southern sector, Heron Island. Associated yearly maximum Degree Heating Weeks (DHW) are provided.

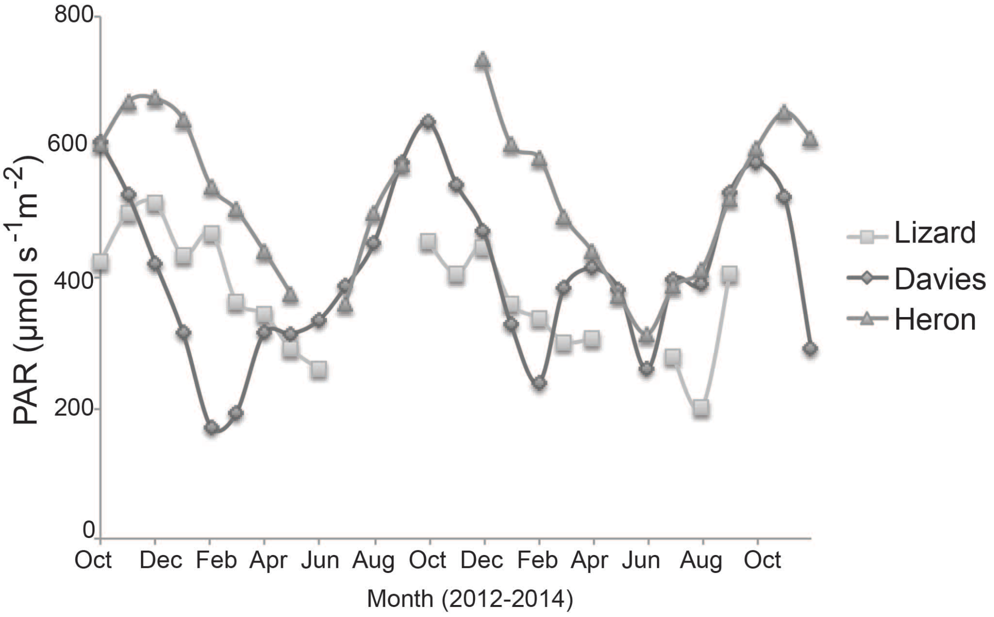

Figure S4. Monthly average solar irradiance (PAR) in the northern (Lizard Island), central (Davies Reef) and southern (Heron Island) sectors of the GBR. Data were determined from IMOS weather stations at each reef[64].

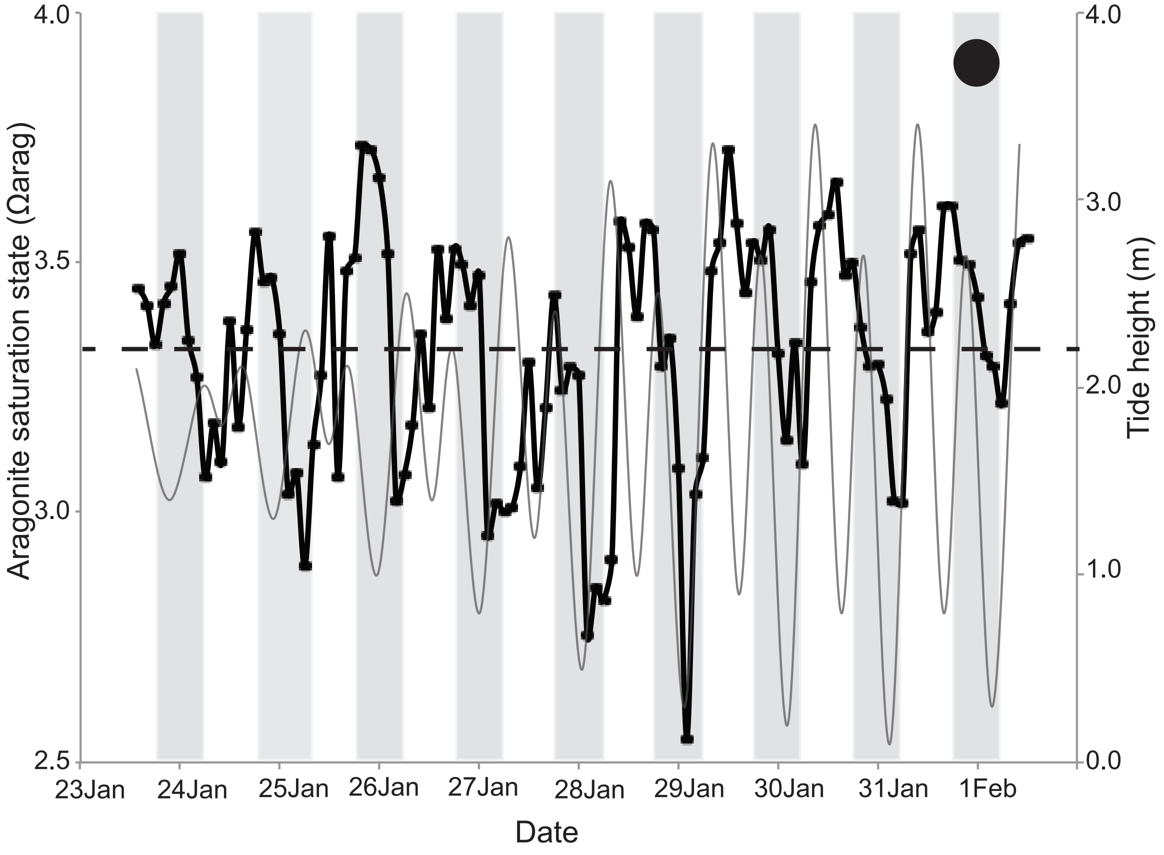

Figure S5. Diel variation of within-reef aragonite saturation at Lizard Island from 23 January to 2 February 2014. Vertical grey shading represents nighttime. Black line is the aragonite saturation and thin grey line represents tide height (m). The horizontal black lines is the average saturation state (3.3).

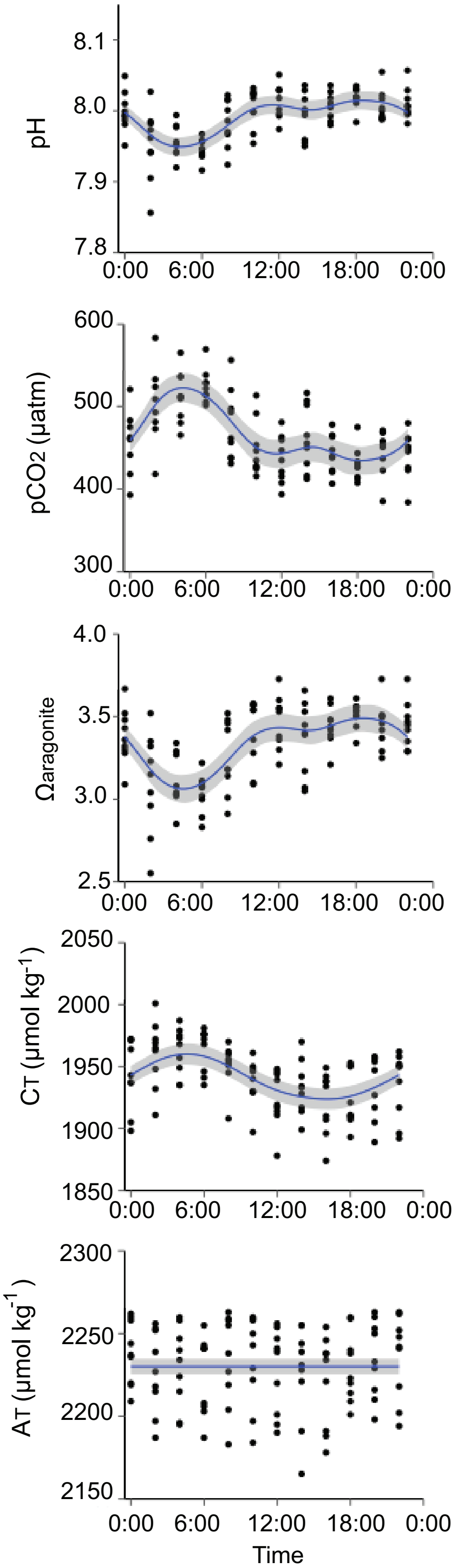

Figure S6. Diel curves of carbonate chemistry parameters during the summer, Jan 2014 at Lizard Island. Data points show data from 9-consecutive days with shaded lines representing 95% confidence intervals. pCO_2= partial pressure of carbon dioxide. C_T= Total inorganic carbon. A_T= total alkalinity.

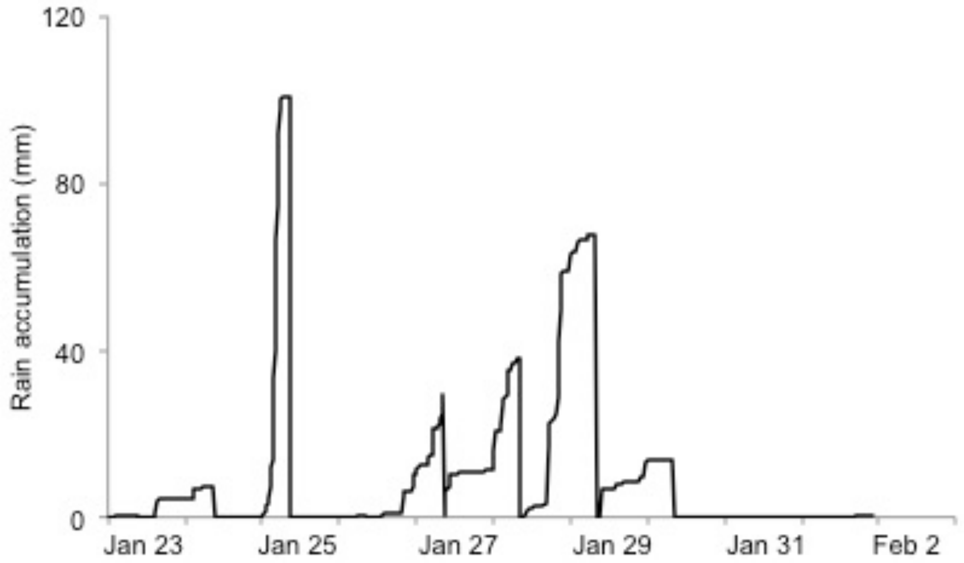

Figure S7. Rain accumulation (mm) at Lizard Island during the course of deployment of the automated water sampler from Jan 23-Feb 2 at Lizard Island. Data were downloaded 25/05/15 from Lizard Island IMOS Weather Station[64].

Supplementary Tables

Table S1. Linear mixed effect model results for linear extension (LE), calcification (Calc) and density (Dens) with associated transformation (Sqrt=square root) of fixed effect sector and nest random effects (Time, Site and Colony). Selected model for each response variable in bold based on lowest AIC.

Model		Response Variable	Fixed Effect	Random Effect	df	AIC
1	*Acropora muricata*	LE	Sector		4	4316
2		LE	Sector	Time	5	4298
3		LE	Sector	Time period/Site	6	4172
**4**		**LE**	**Sector**	**Time period /Site/Colony**	**7**	**4080**
6	*Acropora muricata*	SqrtCalc	Sector		4	569
7		SqrtCalc	Sector	Time period	5	558
8		SqrtCalc	Sector	Time period Site	6	481
**9**		**SqrtCalc**	**Sector**	Time period **/Site/Colony**	**7**	**466**
11	*Acropora muricata*	SqrtDens	Sector		4	-985
12		SqrtDens	Sector	Time period	5	-984
13		SqrtDens	Sector	Time period /Site	6	-994
**14**		**SqrtDens**	**Sector**	Time period **/Site/Colony**	**7**	**-1001**
15	*Isopora palifera*	LE	Sector		4	87
**16**		**LE**	**Sector**	**Colony**	5	72
17		SqrtDens	Sector			-98
**18**		**SqrtDens**	**Sector**	**Colony**		**-99**
19		Calc	Sector			68
20		**Calc**	**Sector**	**Colony**		**67**
21	*Pocillopora damicornis*	SqrtLE	Sector		4	2641
22		SqrtLE	Sector	Time period	5	2642
23		SqrtLE	Sector	Time period/Site	6	2471
24		SqrtLE	**Sector**	**Time period/Site/Colony**	**7**	**2022**
**25**	*Pocillopora damicornis*	**SqrtDens**	**Sector**		**4**	**-386**
26		SqrtDens	Sector	Time period	5	-384
27		SqrtDens	Sector	Time period/Site	6	-382
**28**	*Pocillopora damicornis*	**SqrtCalc**	**Sector**		4	**-229**
29		SqrtCalc	Sector	Time period	5	-227
30		SqrtCalc	Sector	Time period/Site	6	-225

Table S2. Linear mixed effect model results for *Acropora muricata*, *Pocillopora damicornis* and *Isopora palifera* linear extension, calcification and density as the response variable for the fixed effects of sector: northern sector (Lizard Island), central sector (Davies Reef) and southern sector (Heron Island).

Species	Response variable	Fixed Effects	Value	SE	Df	t-value	p-value
A. muricata	LE	(Intercept)	4.420	0.45	302	10.10	-
		Sector: Central	-0.753	0.60	18	-1.250	0.229
		Sector: Southern	-2.010	0.59	18	-3.390	0.003
	Dens	(Intercept)	0.875	0.04	267	87.11	-
		Sector: Central	0.028	0.06	18	0.943	0.358
		Sector: Southern	-0.029	0.05	18	-1.090	0.291
	Calc	(Intercept)	3.700	0.40	271	18.42	-
		Sector: Central	-0.390	0.51	18	-0.759	0.458
		Sector: Southern	-1.500	0.49	18	-3.280	0.004
P. damicornis	LE	(Intercept)	1.040	0.08	1394	24.71	-
		Sector: Central	-0.129	0.11	8	-1.140	0.228
		Sector: Southern	-0.347	0.10	8	-3.270	0.011
	Dens	(Intercept)	1.216	0.03	90	89.59	-
		Sector: Central	-0.037	0.04	2	-1.047	0.298
		Sector: Southern	0.010	0.04	2	0.282	0.778
	Calc	(Intercept)	1.104	0.06	90	43.54	-
		Sector: Central	-0.147	0.07	2	-2.960	0.043
		Sector: Southern	-0.382	0.06	2	-5.680	0.000
I. palifera	LE	(Intercept)	1.639	0.10	80	16.31	-
		Sector: Central	-0.118	0.14	19	-0.850	0.403
		Sector: Southern	-0.281	0.14	19	-1.970	0.063
	Dens	(Intercept)	1.284	0.05	20	66.91	-
		Sector: Central	-0.115	0.06	20	-1.760	0.942
		Sector: Southern	0.041	0.07	20	0.580	0.570
	Calc	(Intercept)	2.692	0.22	20	12.21	-
		Sector: Central	-0.216	0.30	20	-1.070	0.300
		Sector: Southern	-0.209	0.31	20	-0.670	0.511

Table S3. Nested ANOVA results for *A. muricata* comparison of linear extension (LE), density (Dens) and calcification (Calc) for each reef comparing between sampling periods.

Species	Reef	Fixed effect	Nested ANOVA Results
A. muricata	Lizard Island	LE	$F_{3/66}$=10.59, p=0.000
		Dens	$F_{3/66}$ =0.584, p=0.628
		Calc	$F_{3/66}$=7.525, p=0.000
	Davies Reef	LE	$F_{3/45}$=4.625, p=0.005
		Dens	$F_{3/45}$=3.319, p=0.028
		Calc	$F_{3/45}$=4.145, p=0.011
	Heron Island	LE	$F_{3/73=}$4.381, p=0.007
		Dens	$F_{3/69}$=1.734, p=0.168
		Calc	$F_{3/69}$=5.850, p=0.001
P. damicornis	Lizard Island	LE	$F_{1/24}$=8.69, p=0.007
		Dens	$F_{1/24}$=0.94, p=0.334
		Calc	$F_{1/24}$=2.93, p=0.334
	Davies/Trunk Reef	LE	$F_{1/33}$=4.12, p=0.050
		Dens	$F_{1/30}$=1.06, p=0.310
		Calc	$F_{1/30}$=2.97, p=0.102
	Heron Island	LE	$F_{1/33}$=1.56, p=0.221
		Dens	$F_{1/30}$=0.05, p=0.823
		Calc	$F_{1/30}$=0.56, p=0.461

Table S4. Linear regression results for *A. muricata* and *P. damicornis* for the dependent variables (linear extension (cm 6-month^{-1}), density (g cm^{-3}) calcification (g cm^{-2} 6-month^{-1}) and independent variables, sea surface temperature (°C) and light intensity (PAR) averaged from each sampling period and reef.

Species	Independent variable	Dependent Variable	F-value	p-value	R^2
A. muricata	SST (°C)	Linear extension	$F_{1/10}$=5.058	0.048	0.34
		Density	$F_{1/10}$=0.169	0.690	0.02
		Calcification	$F_{1/10}$=5.774	0.037	0.37
P. damicornis		Linear extension	$F_{1/4}$=5.774	0.134	0.46
		Density	$F_{1/4}$=4.782	0.094	0.54
		Calcification	$F_{1/4}$=2.128	0.218	0.35
A. muricata	Light Intensity (PAR)	Linear extension	$F_{1/10}$=2.339	0.157	0.19
		Density	$F_{1/10}$=3.189	0.104	0.24
		Calcification	$F_{1/10}$=2.733	0.129	0.21
P. damicornis		Linear extension	$F_{1/4}$=0.242	0.649	0.06
		Density	$F_{1/4}$=1.791	0.252	0.31
		Calcification	$F_{1/4}$=0.651	0.465	0.14

Table S5. Comparison of the historic annual long-term average SST (1965-2000) to the study annual average (2012-2014) at Lizard Island, Davies Reef and Heron Island using a Student's t-test.

	Historic long-term average SST (°C)	Study average SST (2012-2014) (°C)	t-test results
Lizard Island	25.83 ± 0.07	26.02 ± 0.07	t=0.801, df=37, p=0.428
Davies Reef	25.73 ± 0.06	25.79 ± 0.04	t=0.299, df=37, p=0.767
Heron Island	23.90 ± 0.05	23.96 ± 0.04	t=0.270, df=37, p=0.789

Table S6. Physical conditions during deployment of the automated water sampler at Lizard Island.

	Live Coral	Algae	CCA	$CaCO_3$ substrate-sand, rock, rubble	Other substrate
Mean % ±SE	16.6±2.8	8.8±3.2	1.6±0.4	44.6±7.4	29.9±4.6

Table S7. Values utilised for metadata analysis on linear extension of *A. muricata*. Average linear extension was determined from mean range values when only range provided. *When the year of study was not provided, the year prior to publication was assumed

Location	Date Sampled	Latitude	Depth (m)	SST	Annual extension (cm yr^{-1})	Reference
**Guam**	1977	13.5	3-9	27.04	3.3	65
**Kavaratti atoll, Lakshadweep, India**	1988	10.33	2	28.30	7.9	66
**Kavaratti atoll, Lakshadweep, India**	1989	10.33	2	27.85	8.2	66
**Phuket Thailand, Nai Yang**	1984	8	-	28.45	11.8	67
**Phuket Thailand, S. Bangtoa**	1984	8	-	28.45	9.4	67
**Phuket Thailand, Kamala**	1984	8	-	28.45	14.1	67
**Phuket Marine Biological Center (PMBC)**	1981	8	3	28.52	8.0	68
**Sri Lanka, Hikkaduwa**	1997	6.14	0.5-1.5	27.86	11.8	69
**Sri Lanka, Roomassala**	1997	6.01	0.5-1.5	27.72	12.1	69
**Maldives**	2010	4.18	1	28.62	5.85	70
**Middle Reef**	2009	-12.5	1-3	27.04	6.3	71
**Lizard Island, GBR**	1984*	-14.66	2	25.93	7.13	72
**Lizard Island, GBR**	2012-13	-14.66	5	26.01	7.93	73
**Lizard Island, GBR**	2013-14	-14.66	5	26.15	10.8	73
**Davies Reef GBR**	1980	-18.51	5	26.02	8	55
**Davies Reef GBR**	1980	-18.51	10	26.02	12.4	55
**Davies Reef GBR**	1980	-18.51	15	26.02	16.6	55
**Davies Reef GBR**	2012-13	-18.51	5	26.0	7.34	73
**Davies Reef GBR**	2013-24	-18.51	5	26.1	7.93	73
**Nelly Bay, GBR**	1980	-19.1	-	26.13	8.82	54
**Dampier Archipelago**	1982	-20.53	-	27.04	13.7	74
**Heron Island, GBR**	2012-13	-23.44	5	23.88	3.93	73
**Heron Island, GBR**	2013-14	-23.44	5	23.99	4.57	73
**Houtman Arbolhous**	1979	-28.7	2-3	21.65	4	75
**Houtman Arbolhous**	1984	-28.7	10	22.14	7.6	76
**Houtman Arbolhous**	1984	-28.7	7	22.14	6.56	76
**Houtman Arbolhous**	1984	-28.7	8	22.14	5.86	76
**Houtman Arbolhous**	1984	-28.7	10	22.14	5.03	76

Table S8. Values utilised for meta-analysis on linear extension of *Pocillopora damicornis*. Average linear extension was determined from mean range values when only range provided. *When the year of study was not provided, the year prior to publication was assumed. ETP=Eastern Tropical Pacific

Location	Ecoregion	Year of study	Lat	Depth (m)	SST	LE (cm yr^{-1})	Reference
**Na'ama Bay, Egypt**	IndoPacific	1998	27.9	5	25.4	0.74	77
**Na'ama Bay, Egypt**	IndoPacific	1998	27.9	15	25.4	0.66	77
**Kaneohe Bay, Oahu**	IndoPacific	1987	21.45	-	24.6	1.64	78
**Hawaii**	IndoPacific	1972	19.7	-	24.4	1.35	79
**Guam**	IndoPacific	1976	13.26	3-9	27.8	2.90	65
**Enewotak Atoll, Marshall Is**	IndoPacific	1972	11	-	27.5	2.64	80
**Enewotak Atoll, Marshall Is**	IndoPacific	1980	11	-	27.6	2.50	81
**Palmitas, Costa Rica**	ETP	1996	10.67	3-5	27.3	5.31	82
**Huevos, Costa Rica**	ETP	1991	10.64	3-5	27.5	6.68	82
**San Pedrito, Costa Rica**	ETP	1996	10.5	3-5	27.2	3.80	82
**Secas Is, Panama**	ETP	1974	8.98	3	25.4	3.96	83
**Secas Is, Panama**	ETP	1974	8.98	6	25.4	3.36	83
**Secas, Gulf of Chiriqui**	ETP	1971	8.98	-	26.0	3.86	84
**Pacific Panama**	ETP	1980	8.97	-	26.4	4.80	81
**Isla Contradora, Panama**	ETP	1978	8.63	1	26.5	5.43	85
**Isla Contradora, Panama**	ETP	1978	8.63	7	26.5	4.61	85
**Saboga Island, Gulf of Panama**	ETP	1971	8.61	-	27.7	3.08	84
**Cano Island, Costa Rica**	ETP	1985	8.43	2-3	27.4	3.46	86
**Cano Island, Costa Rica**	ETP	1985	8.43	8-10	27.4	2.98	86
**Pearl Island (Gulf of Panama)**	ETP	1971	8.39	2-4	26.4	4.20	89
**Uva Reef, Panama**	ETP	2003	8	2-3	28.0	2.78	88
**Ko Phuket, Thailand**	IndoPacific	1983	7.53	-	28.8	1.43	89
**Galapagos**	IndoPacific	1978	- 0.49	1-4	22.5	2.24	90
**Lizard Island, GBR**	IndoPacific	2013	-14.66	5	26.2	2.17	73
**Lizard Island, GBR**	IndoPacific	1984	-14.68	-	25.8	3.66	72
**Davies/Trunk Reef GBR**	IndoPacific	2013	-18.51	5	25.9	1.97	73
**Palm Island, GBR**	IndoPacific	1984	-18.74	-	25.3	4.32	72
**Dampier Archipelago**	IndoPacific	1982	-20.53	-	25.8	4.50	74
**Heron Island, GBR**	IndoPacific	2013	-23.44	5	24.0	1.47	73
**Houtman Arbolhous**	IndoPacific	1979	-28.72	2-3	21.5	1.33	75
**Solitary Islands**	IndoPacific	1994	-30	-	22.1	1.24	91
**Lord Howe Island**	IndoPacific	2010	-31.3	4	21.7	1.16	47
**Lord Howe Island**	IndoPacific	1993	-31.5	3	21.1	1.61	91
**Rottnest Island, WA**	IndoPacific	1989	-32	-	20.7	1.50	92
**Rottnest Island, WA**	IndoPacific	1989	-32	-	20.7	0.90	92

References for Table S7 and S8

65. Neudecker, S. Growth and survival of scleractinian corals exposed to thermal effluents at Guam. *Proc 4th ICRS, Manila* **1**, 173-180 (1981).

66. Suresh, V. R. & Mathew, K. J. Skeletal extension of staghorn coral *Acropora formosa* in relation to environment at Kavaratti atoll (Lakshadweep). *Indian J Mar Sci* **22**, 176-179 (1993).

67. Changsang, H., Phongsuwan, N. & Boonyanate, P. Growth of corals under effect of sedimentation along the northwest coast of Phuket Island, Thailand. *Proc 7th ICRS, Guam* **1**, 241-248 (1992).

68. Charuchinda, M. & Chansang, H. Skeleton extension and banding formation of *Porites lutea* of fringing reefs along the south and west costs of Phuket Island (Thailand). *Proc 5th ICRS, Tahiti* **6**, 83-87 (1985).

69. Jinendradasa, S. S. & Ekarame, S. U. K. Linear extension of *Acropora formosa* (Dana) at selected reef locations in Sri Lanka. *Proc. 9th ICRS., Bali, Indonesia* **1**, 23-27 (2000).

70. Morgan, K. M. & Kench, P. S. Skeletal extension and calcification of reef-building corals in the central Indian Ocean. *Mar Environ Res* **81**, 78-82, doi:http://dx.doi.org/10.1016/j.marenvres.2012.08.001 (2012).

71. Browne, N. K. Spatial and temporal variations in coral growth on an inshore turbid reef subjected to multiple disturbances. *Mar Environ Res* **77**, 71-83, doi:http://dx.doi.org/10.1016/j.marenvres.2012.02.005 (2012).

72. Oliver, J. An evaluation of the biological and economic aspects of commercial coral collecting in the Great Barrier Reef region. 106 (Final report to the Great Barrier Reef Marine Park Authority, 1985).

73. This study

74. Simpson, C. J. *Ecology of scleractinian corals in the Dampier Archipelage, Western Australia*. Vol. 23 1-238 (Environmental Protection Authority, 1988).

75. Crossland, C. J. Seasonal growth of *Acropora cf. formosa* and *Pocillopora damicornis* on a high latitude reef (Houtman Abrolhos, Western Australia). *Proc. 4th ICRS,, Manila* **1**, 663-667 (1981).

76. Harriott, V. J. Growth of the staghorn coral *Acropora formosa* at Houtman Abrolhos, Western Australia. *Mar Biol* **132**, 319-325 (1998).

77. Kotb, M. M. A. Growth rates of three reef-building coral species in the northern Red Sea, Egypt. *Egyptian J Aqua Biol Fish* **5**, 165-185 (2001).

78. Romano, S. L. Long-term effects of interspecific aggression on growth of the reef-building corals *Cyphastrea ocellina* (Dana) and *Pocillopora damicomis* (Linnaeus).

J Exp Mar Biol Ecol **140**, 135-146, doi:http://dx.doi.org/10.1016/0022-0981(90)90087-S (1990).

79. Maragos, J. E. *A study of the ecology of Hawaiian reef corals* PhD dissertation thesis, University of Hawaii, (1972).

80. Stimson, J. The effect of shading by the table coral *Acropora hyacinthus* on understory corals. *Ecology* **66**, 40-53, doi:10.2307/1941305 (1985).

81. Richmond, R. H. Energetic relationships and biogeographical differences among fecundity, growth and reproduction in the reef coral *Pocillopora damicornis*. *Bull Mar Sci* **41**, 594-604 (1987).

82. Jimenez, C. & Cortes, J. Growth of seven species of scleractinian corals in an upwelling environment of the eastern Pacific (Golfo de Papagayo, Costa Rica). *Bull Mar Sci* **72**, 187-198 (2003).

83. Glynn, P. W. Some physical and biological determinants of coral community structure in the Eastern Pacific. *Ecolog Monographs* **46**, 431-456 (1976).

84. Glynn, P. W. Coral growth in upwelling and non-upwelling areas off Pacific coast of Panama. *J Mar Res* **35**, 567-585 (1977).

85. Wellington, G. M. An experimental analysis of the effects of light and zooplankton on coral zonation. *Oecologia* **52**, 311-320, doi:10.1007/bf00367953 (1982).

86. Guzmán, H. M. & Cortes, J. Growth rates of eight species of scleractinian corals in the Eastern Pacific (Costa Rica). *Bull Mar Sci* **44**, 1186-1194 (1989).

87. Glynn, P. W. & Stewart, R. H. Distribution of coral reefs in the Pearl Islands (Gulf of Panama) in relation to thermal conditions. *Limn Oceanogr* **18**, 367-379 (1973).

88. Manzello, D. P. Coral growth with thermal stress and ocean acidification: lessons from the eastern tropical Pacific. *Coral Reefs* **29**, 749-758, doi:10.1007/s00338-010-0623-4 (2010).

89. Martin, D. A. & Le Tissier, A. The growth and formation of branch tips of *Pocillopora damicornis* (Linnaeus). *J Exp Mar Biol Ecol* **124**, 115-131, doi:http://dx.doi.org/10.1016/0022-0981(88)90115-3 (1988).

90. Glynn, P. W., Wellington, G. M. & Birkeland, C. Coral reef growth in the Galapagos: limitation by sea urchins. *Science* **203**, 47-49, doi:10.2307/1747555 (1979).

91. Harriott, V. J. Coral growth in subtropical eastern Australia. *Coral Reefs* **18**, 281-291 (1999).

92. Ward, S. Two patterns of energy allocation for growth, reproduction and lipid storage in the scleractinian coral *Pocillopora damicornis*. *Coral Reefs* **14**, 87-90, doi:10.1007/bf00303428 (1995).
